# Design and analysis of TwinCardio framework to detect and monitor cardiovascular diseases using digital twin and deep neural network

**DOI:** 10.1038/s41598-025-08824-3

**Published:** 2025-07-08

**Authors:** A. Anandita Iyer, K. S. Umadevi

**Affiliations:** Vellore Institute of Technology, Vellore, TN 632014 India

**Keywords:** Digital twin, Cyber-physical system, Cardiovascular disease, Internet of Things, Healthcare, Cardiology, Health care, Engineering

## Abstract

World Health Organization (WHO) estimates 17.9 million deaths globally every year due to Cardiovascular Disease or CVD, which includes an array of disorders of the heart and blood vessels, that includes coronary heart disease, cerebrovascular disease, rheumatic heart disease, and various other conditions. Notably, there has been nearly 30% increase in heart attack cases among individuals aged 25–44 between 2020 and 2023. These alarming trends make it pertinent for a deeper comprehensive integration of precision healthcare with digital twin. With the development of technologies, such as machine learning, cyber-physical systems, and the Internet of Things (IoT), digital twin is being applied in various industries as a precision simulation technology from concept to practice. Combining healthcare with digital twin paves the path to a more efficient means of delivering accurate and timely services to patients suffering from heart diseases. However, achieving personalized and precise healthcare management requires humans to be in loop with the digital twin, which will facilitate the integration of the patient’s physical world with the medical virtual world to realize smart healthcare. This work proposes “TwinCardio”—a novel reference framework of digital twin enabled smart health monitoring and “TwinNet”—a customized neural network designed for cardiovascular disease classification and prediction. TwinCardio framework is designed for patient monitoring, diagnosing and predicting the aspects of the health of individuals using on-body sensors. It depicts different layer that describes continuous data acquisition, data simulation, evaluation inline with security protocols thus serving as a base to manufacture smart healthcare models.

## Introduction

The digitization of healthcare has emerged as a transformative force since the late twentieth century, driven by technological innovations, governmental initiatives, and a pressing need for improved efficiency and patient care. Although early examples of computerized medical records, such as the Medical Data Language System (MEDLAS) developed by the University of Massachusetts, existed, the widespread adoption of Electronic Health Records (EHRs) began in 1990s and accelerated in 2000s. This shift marked a pivotal moment in the transition from traditional healthcare models to more digitized approach. In conjunction with EHRs, advancements in medical imaging technologies, telemedicine, and health applications further reshaped healthcare delivery, enhancing access, coordination, and efficiency in both clinical and administrative processes^1^. The growth of networking infrastructure, increased data storage capacities, and faster data analysis capabilities have been crucial in enabling the digitalization of healthcare. These advancements facilitate the collection, storage, and analysis of large volumes of medical data, allowing healthcare providers to detect abnormalities and patterns that might be otherwise undetectable with traditional methods. As a result, digital technologies have contributed significantly to reducing medical errors, improving patient outcomes, and enhancing treatment efficiency^2^. Central to these developments is the role of EHRs, which provide healthcare professionals with real-time access to patient records, regardless of location or time, thereby bridging gaps in care coordination^3^. The integration of Artificial Intelligence (AI) into healthcare practices further advances diagnostic and treatment planning, allowing for more personalized and efficient care. Additionally, the incorporation of Internet of Things (IoT) devices, such as wearable sensors, in conjunction with EHRs, supports continuous health monitoring, offering new possibilities for personalized treatment plans and proactive health management^4^.

Wearable devices—such as smartwatches, fitness trackers, and glucose monitors—continuously collect data on vital signs like heart rate, blood pressure, and glucose levels. This data is transmitted to healthcare providers, enabling them to detect early signs of conditions such as arrhythmia or hypertension. Moreover, wireless data transmission from implanted devices, such as pacemakers or defibrillators, provides valuable insights into a patient’s cardiac health. Advanced imaging technologies, including MRI and CT scans, also contribute to more accurate diagnoses and serve as crucial tools for monitoring patient conditions over time^5^. Furthermore, the digitalization of healthcare has enabled the development of remote patient monitoring systems, which allow for continuous patient observation from the comfort of their homes. This approach is particularly beneficial for elderly patients, those with chronic conditions, and individuals residing in remote areas where frequent hospital visits may not be feasible^6^.

**Fig. 1:**
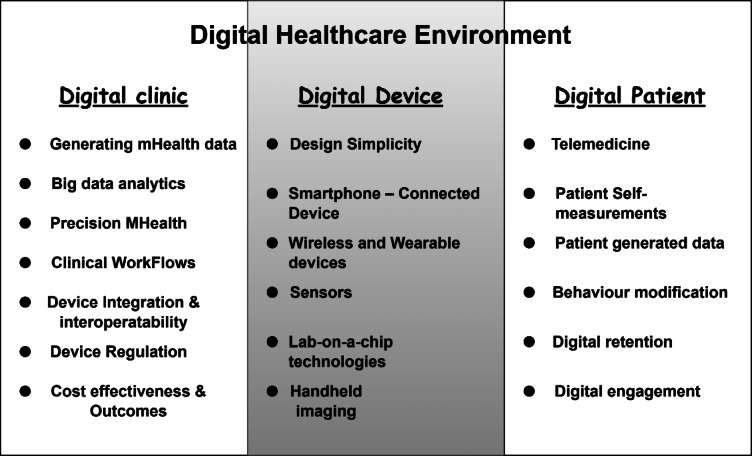
Digitalization of different aspect of healthcare^9^.

An emerging area within the digital transformation of healthcare is the use of digital twin technology. By leveraging data from IoT devices and sensors, digital twins can create accurate, real-time simulations of patients, medical equipment, or healthcare environments. These simulations provide healthcare professionals with powerful tools for diagnosis, treatment planning, and predictive maintenance of medical systems. Despite its success in industrial applications, the integration of digital twins in clinical practices has been slower, though it holds tremendous potential to revolutionize patient care and hospital management^7^. This article explores the ongoing digitalization of healthcare, with a particular focus on the impact of EHRs, IoT devices, AI, and digital twins in shaping the future of medical practice. As healthcare continues to evolve from a reactive to a proactive model, these technologies offer the promise of improved patient care, more efficient resource management, and enhanced healthcare outcomes. However, challenges remain in fully realizing the potential of these advancements in clinical settings^8^. Figure 1 clearly depicts the landscape of digitalization in a healthcare environment.

This paper proposes a reference framework to apply Digital Twin to assess cardiac health in patient care called TwinCardio. The proposed framework is used to monitor patients suffering from cardiovascular diseases and suggests various services such as real-time cardiac health monitoring and prediction. Otherwise unchecked, the current health status could lead to any future complications^10^. Further, the work designs a custom neural network model called TwinNet, for classification of cardiovascular disease. The proposed reference framework of the TwinCardio environment that contains an IoT device (sensor) along with edge computation is constructed. Finally, the paper discusses briefly on how TwinCardio enables personalized patient care.

## Understanding key concepts

### Chronic disease

A chronic disease is an ailment but they are not transmissible, usually lasts for a long time and grows gradually due to poor lifestyle choices, environmental factors and/or genetics. It was observed that, chronic diseases caused around 28 million deaths across the world in 1990, or 57% of the total number. This number grew to about 36 million (63%) of all fatalities in 2008 and 39 million (72%) of all deaths in 2016. Although life expectancy has increased over the past two centuries, the current estimates suggest that future generations may experience a decline in life expectancy due to the rise in chronic diseases like obesity, cardiovascular disease, lower respiratory disease, cancer and stroke. The World Health Organization (WHO) reports that heart disease causes 12 million deaths globally each year. In total, 17.9 million people die from heart disease, which accounts for about 31% of deaths globally. WHO predicts that by 2030, heart disease might increase the death toll to nearly 23.6 million. The vast amount of data that has been generated by WHO to predict heart disease is too complex for conventional methods to analyze and interpret patient health condition effectively. However, using data processing tools, disease prediction can be more accurate in considerably less time^11^. Therefore, the aim of the TwinCardio model is to analyze, detect, and predict cardiovascular diseases. Table 1 depicts the evolution of various scanning, imaging and detection technologies of cardiovascular diseases and brief comparison has been drawn between the past, current and future trends.

**Table 1:** Evolution in digital technologies to detect CVD.

Technologies	Past methods	Current trends	10 year future prediction
Cardiac rhythm monitoring^12^	Surface ECG Holter monitor ICD/pacemaker	iECG telemetry patches—smartwatches leadless implantable devices	Contactless rhythm monitoring multilead iECG electronic-epicardium
Hemodynamic monitoring^13^	Blood pressure cuff pulmonary artery catheter	Implantable intracardiac devices wireless-invasive hemodynamic monitoring blood pressure watches	Blood pressure nanosensors wearable blood pressure patches
Myocardial structure^14^	Endomyocardial biopsy	Breath & genetic markers	Portable optical imaging technologies
Imaging myocardial structure^15^	Traditional echocardiography myocardial perfusion imaging	Handheld echocardiography hybrid imaging	Circulating ultrasound-nanochips robotic ultrasonography
Imaging myocardial function^15^	Doppler & color flow imaging	3D–4D echocardiography molecular imaging	Holographic imaging cloud-based imaging platforms big-data cognitive computing analytics
Cardiac valves^16^	Bioprosthetic & mechanical valves	Transcatheter valve implantation valve ‘clips’	3D printed tissue valves sensor-based cardiac regenerative therapy
Medication profiling^17^	Pill-counting patient recollection	Smartphone-connected pill bottles wireless-observed therapy	Gene-based nanosensors for targeted therapeutics wireless drug delivery microchips ultrasound therapeutics
Metabolic, genomic & proteomic profiling^18^	Traditional laboratory testing	Transcutaneous sensors smartphone laboratory testing handheld genomic sequencers	Lab-on-a-chip-lab-on-skin medical-tricorders breath-o-mics-breath biomarkers on handheld devices

### Cardiovascular disease (CVD)

The term “cardiovascular disease” refers to a wide spectrum of disorders that impacts the heart and blood arteries. Due to various factors, like aging populations, sedentary lifestyles, and bad diets, CVD is becoming more common and is one of the top causes of mortality globally. Few major causes of CVD are *atherosclerosis*, the buildup of plaque in the arteries composed of cholesterol, fatty substances etc., that can narrow the arteries reducing blood flow and increasing the risk of blood clots; *inflammation* plays a crucial role in the development and progression of CVD and chronic inflammation can damage the lining of the arteries, making them more susceptible to plaque buildup; *high blood pressure* strains the heart and blood vessels thereby increasing the risk of CVD, over time damages heart muscle leading to heart failure^19^.

TwinCardio aims to identify the following CVDs:

Cardiomyopathy—Heart muscle disease weakening heart’s ability to pump blood effectively. It can be caused by the enlarged left ventricle or sometimes due to stretched muscles of the heart wall^20^.

Arrhythmia—An irregular heartbeat occurs due to change in timing or pattern of the heart’s rhythm which can cause the heart to beat too fast (tachycardia), too slow (bradycardia), or irregularly^21^.

Cardiomegaly—The enlargement of the heart occurs when the muscles become thicker than usual, or it is stretched more than 50%, therefore causing difficulty in pumping blood. This condition usually occurs when the patient has had a heart attack or the heart weakens, it begins to enlarge thus forcing heart to work harder to pump the blood^22^.

The primary challenges in healthcare industry faced today includes aging, lack of medical professionals and increase in overall cost of healthcare. In 2013, the World Health Organization (WHO) reported that there was a need for 60.4 million health workers worldwide, while the actual number of healthcare workers was 43 million. By 2030, both numbers will rise to 81.8 million and 67.3 million, respectively. As a result, the severe medical staffing deficit is still unresolved. The likelihood of contracting diseases, illnesses, and the need for ongoing care and medical treatments increases with age. The birth rate has been low in the past and is predicted to continue low in the upcoming decades, which is one of the key causes. By 2050, the proportion of population over 60 getting affected will double from 11 to 22%. As a result, the healthcare services provided for the age group of 60 and older should receive additional funding and human resources. A significant amount of the gross domestic product (GDP) has gone toward healthcare spending overall. According to the WHO survey, countries majorly affected includes India, China, the Russian Federation, Brazil, Australia, Canada and the United States are 4.7%, 5.6%, 7.1%, 8.3%, 9.4%, 10.5% and 17.1% respectively^23^. These numbers will rise over the next few decades simply because of aging.

The good news is that healthcare applications can now incorporate artificial intelligence, resulting in smart healthcare, owing to the quick rise in processing power and the accessibility of health data. In addition to promoting sustainable development, this can undoubtedly aid in resolving some of the healthcare issues described above. The goal of healthcare sustainability is to maximize the health service’s social and economic effects while maintaining patient health and its ability to provide healthcare in the future. In reality, a higher percentage of the elderly and a shortage of medical workers result in higher government expenditures and more dire social productivity^24^.

To facilitate the integration of digital world along with patient data and HER, TwinCardio integrates Digital Twin and Cyber-Physical System to create a real-time simulation model of patient heart which in turn will help explore the aforementioned diseases and the extent of the diseases.

### Digital twin

Digital Twin was first categorized into three dimensions: physical (product in physical environment), virtual (digital representation of physical model in virtual space) and connection (information flow between both physical and virtual space), where it can be seen that the physical and virtual space is connected by an entity that promotes the exchange of information^25^. Digital Twin can be defined as computer model or machine-based model which helps in simulating and mirroring the lifecycle of a physical object, human or any process or feature. Each twin is connected to its physical model with the help of a unique key. These keys identify the relationship between a physical object with its virtual model, hence making their relationship bijective. A digital twin should not be mistaken as a simple model or just a simulation^26^. It is an intelligent, ever evolving digital model of a physical process. It effortlessly follows its physical model and its lifecycle in order to monitor the process, control its development and optimize the functions. It works by predicting the events that might take place in future, machine status, possible failures and damages and allows testing new configurations, perform simulations. It is a closed-loop optimization process, continuously synchronizing physical entity and its surrounding with the virtual twin. There exists a continuous real time interaction between physical and digital twin as a consequence the digital replica is always aware of its physical world. This communication along with data analytics algorithms, data fusion, proceeded by AI and ML algorithms helps the digital twin to better grow with its physical environment rather than being just a simulation and thus providing scope for faster reconfiguration. It is then continuously updated according to the conditions of the physical twin and the traits of the twins are governed. As the digital twin is compiled with AI, it can easily identify and uncover system-related information, hidden patterns and unknown system correlations^27^.

Apart from data modelling, another equally important feature is prediction. The ability of digital twin is to predict the performance of different processes, probable ways to increase system accuracy to avoid failures and damages. It is possible by the integration of digital twin with advanced data analytics and artificial intelligence^28^. As digital twin is integrated with AI, there exist a two-way flow of information. The data is captured from physical environment using sensors based on which a digital replica is created mimicking the behaviour of the physical entity. The twin which is shared with the intelligent system that will predict the future outcomes which then can be shared with the real entity in the form of feedback. Hence, this dynamic information flow between physical world and virtual model helps in creating a complex yet powerful digital representations of the real-world object, which will then pave a path towards in depth testing, better decision making without disturbing or tampering the physical entity.

A Healthcare Digital Twin refers to a virtual representation of a physical patient, organ, system, or clinical environment that is continuously updated with real-time data and used for simulation, monitoring, and predictive analysis. It integrates data from multiple sources such as electronic medical records (EMRs), wearable sensors, imaging, lab results, genomics, and lifestyle information to create a dynamic computational model that mirrors the actual state and behaviour of a patient or healthcare system. This digital replica mirrors the physiological and clinical state of the real-world entity and enables simulation, monitoring, and predictive modelling. Unlike static models, a healthcare digital twin evolves with the patient, using artificial intelligence and machine learning algorithms to refine its predictions and insights over time. It allows clinicians to test “what-if” scenarios, evaluate treatment outcomes, and develop personalized care strategies by providing a safe, data-driven environment for decision support. Digital twins are increasingly being applied in areas such as cardiology, oncology, and hospital operations to enhance diagnostic accuracy, optimize resource management, and improve patient outcomes through precision medicine^29^.

### Cyber-physical systems

Cyber Physical System (CPS) are complex and multidimensional system that combine ever changing physical world with cyber world. CPS provides information feedback, control and services by integrating computing, control and communication (3C). The prominent role of CPS is to control and simulate and integrate the physical processes with computational processes, whereas a digital twin talks mostly about creating a virtual image of physical entity. It simulates the real-world behaviour via its digital replica and bi-directional. One major difference between CPS and digital twin is that, CPS is inclined towards science whereas digital twin is towards technology. But both concepts work towards smart factory and smart manufacturing^30^.

The digital twin works with CPS by following the mechanism of closed loop feedback, where it integrates with/within process achieved during implementation by creating a virtual model of the physical entity and mirroring entire behaviours of the real-world object to the replica and providing feedback to achieve control. This control loop works by first collecting the data from sensors like, process information, current state etc. and sending it to the virtual model, which then sends feedbacks and commands back to the physical object hence closing the control loop^31^. In this way, the industrial process and machines, turns into an enhanced objects which will become self-sufficient, self-reliant and swiftly respond to changes. Thus, making cyber-physical integration possible and easy to monitor, control, optimize and even make accurate predictions of future behaviours.

## Literature survey

Digital twin in healthcare monitoring is considered as a revolutionary method to provide better and more personalized healthcare services to patients. In general, reports on digital twins applied to healthcare are grouped as technology for precise diagnosis, personalized healthcare and challenges that remains in deployment of digital twin framework. This section reviews the existing work and studies on different technologies and understand the challenges and effective implementation of personalized patient care using digital twin.

A novel approach for diagnosing Hypertrophic Cardiomyopathy (HCM) using deep learning is presented, in comparison with conventional techniques, and shows enhanced detection accuracy using convolutional neural networks (CNNs) to evaluate electrocardiogram (ECG) data^32^. The model was trained on a sizable dataset and exhibits the potential for non-invasively detecting HCM—a disorder that frequently remains undetected because of its mild clinical symptoms. The findings suggests that the CNN-enabled ECG analysis can serve as an important means of early diagnosis, leading to quick interventions and better patient outcomes. However, detailed and diverse demographic validation is required to establish an extensive application of the work. Under the same umbrella of concept, the work in^33^ combines the advantages of linear models and random forest to identify cardiovascular disease. The RERF-ILM algorithm, integrates the interpretability and simplicity of linear models with the recursive feature selection of random forest to design an algorithm that produces enhanced prediction accuracy. The Internet of Medical Things (IoMT) is used in the study to gather real-time data from wearable medical sensors. A significant improvement is recorded in cardiovascular disease detection accuracy therefore, it can be said that the proposed algorithm works well with large volumes of medical data and performs quick decision-making in critical scenarios. However, the paper also notes challenges in real-time deployment, such as data privacy concerns and computational efficiency, which needs further exploration.

The work in^34^ presents an innovative approach to integrate the hospital management environment with a digital twin using Discrete Event Simulation (DES). It integrates ubiquitous technologies like sensors and IoT devices with the hospital twin framework to design a real-time, dynamic simulation of hospital operations. The digital twin analyses patients, performs continuous monitoring, operational efficiency, and resource utilization, thus assisting the hospital staff in making data-driven decisions, hence improving patient care, and enhancing the performance of the model. The work highlights the advantage of integrating DES with ubiquitous computing technologies therefore optimizing hospital workflows, increasing response time in cases or emergencies, and predicting bottlenecks. Velazquez and Alejandro^35^ explores the design framework for a personalized digital twin model, which intends to enhance individual’s well-being experience. A comprehensive ecosystem is suggested incorporating different communication technologies along with numerous data sources, which creates a personalized virtual health model tracking a person’s habits, health, and lifestyle. The author highlights the importance of user-centric design approaches, stating the need to consider the privacy of patient data, data accuracy during the collection as well as user engagement. The work presented explores the benefits, such as improvised healthcare and accurate patient monitoring, as well as the challenges associated, which include technical complexity and ethical issues. The work in^36^ proposed a conceptual methodology that explores the benefits of a digital twin framework in cancer care, and a detailed overview of how a digital twin model can integrate patient genetic data, clinical and imaging data to tailor a personalized treatment plan has been discussed. Additionally, the work presents a scenario analysis to illustrate the potential applications and benefits of digital twin in oncology. A digital twin framework performing Predictive simulation to enhance the hospital-patient pathway is introduced^37^. The work presents a system that can model patient medical courses using real-time data monitoring and simulation techniques, helping hospitals to optimize workflow and patient management. The HospiT’Win, aims to improve hospital resource allocation, reduce wait times, and enhance overall efficiency in a hospital environment by predicting patient requirements, continuous patient monitoring and predicting potential bottlenecks. It highlights the proposed model’s potential to transform any hospital operation but also emphasizes the challenges that occurs in real-time, including data integration and the need for more accurate simulation models.

The paper^38^ explores the integration of the Internet of Things (IoT) and digital twin technology in healthcare systems to enhance context-aware capabilities in analysiing and monitoring patient data. The work proposed by integrating IoT devices to sense/collect real-time data along with a virtual, dynamic model of the patient (in other words—digital twin of the patient) to diagnose the patient in real-time and gather data in context with changing conditions. The integration aims to provide enhanced personalized care to the patient by ensuring more accurate, context-sensitive insights into patient health. The author discusses the benefits of the approach, which includes enhanced patient monitoring, responsive care, and better decision support, while also discussing the challenges related to system interoperability, data integration, and data privacy. Overall, a compelling vision of IoT-enabled digital twin model facilitates intelligent and adaptive healthcare solutions.

The concept of the ‘Dynamic Digital Twin’ model with reference to personalized healthcare, a continuous and real-time virtual model of a patient that slowly evolves over time is explored^39^. The author suggests that the dynamic digital twin technology has the potential to transform the healthcare industry by facilitating personalized treatment plans, accurate diagnosis, and real-time monitoring as this technology evolves with the patient’s ever-changing conditions. Dynamic Digital Twin model will be able to analyse and detect possible diseases occur based on the historical and current records that are continuously gathered from different sources (including real-time sensors, genetic information, etc.) and, therefore, provide individualized preventive methods. In addition, it discusses the challenges that arise during implementation of this technology, which entails data integration, processing power, as well as ethical concerns like privacy and security issues. Though it presents a theoretical concept, it still offers a peek into the fascinating vision of the future of personalized medicine. Over the course of a person’s lifetime, it could transform healthcare from reactive to proactive and preventive care. Another research that examines the transformational power of digital twin in healthcare is discussed in^40^. The author explores the application of digital twin in patient lifestyle monitoring, genetic analysis, diagnosis of disease as well as prediction and personalized healthcare plans. The author focuses on the practical implementation issues like the requirement of standardized data integration, multiple source data collection in real-time, data privacy, substantial amount of computing power, etc., in the design of a patient digital twin model as well as offers a thorough review of technologies like Artificial Intelligence (AI), big data analytics and biosensors that has made the development of digital twin possible. While the patient digital twin technology is still in its infancy, the author argues that the potential this technology has to completely transform healthcare. In the case of healthcare, data security and patient privacy, regulatory barriers and moral ramifications continues to be a major challenge in customizing patient digital twin models. However, if these challenges are solved, there is a likely positive outcome on the impact of digital twin in personalized healthcare in the future. With more emphasis on the predictive power of digital twin, application of digital twin for patient care on the basis of better decision-making, accurate analysis, and reliable health prediction is explored in^41^. The work presents a different point of perspective by arguing that the digital twin model cannot just predict possible diseases but with enough real-time data; it can also predict a person’s behaviour and well-being by including data from wearable sensors, medical records, and social behaviour. This study opens a window towards psychologically analysing a person and make predictions based on behaviours. The paper also covers critical topics such as data protection, ethical dilemmas, and the psychological effects of depending too much on digital prediction. Authors cautions that even though digital twin has the potential to become extremely precise and provide highly customized results, resolving the aforementioned issues is vital to ensure safe use of the technology. Similarly, a study in^42^ examines the development of the human digital twin and the challenges it faces, such as accuracy privacy concerns, data integration hurdles, etc. The survey highlights the upcoming wave of human digital twin and its benefits with health monitoring but also notes that the wider implementation of the technology requires further advancements and proper regulatory considerations in place. The paper^43^ discusses digital twin in advanced precision cardiology by creating a personalized virtual model of a patient’s cardiovascular system. The author discusses how medical imaging, scans, reports, genetic data, and real-time monitoring help improve the diagnosis of the patient, as well as assist the doctor in developing a customized care plan for the patient and generating predictive analysis results based on the data gathered. The study emphasizes the power of digital twin to revolutionize cardiovascular care and also recognizes that in order to achieve the goal of a seamless model, issues like data integration, model validation and privacy concerns must be resolved.

The work in paper^44^ examines the need to build a digital twin model from healthcare point of view, as a healthcare digital twin model not only creates a virtual replica of the human body and/or the hospital environment but it also mimics a person’s physical, biological and behavioural traits. The author discusses various challenges involved in developing a healthcare digital twin model, such as practical, ethical, and technological challenges. These challenges include the collection and integration of real-time data from numerous sensors, advanced visualization and generating AI models based on the data collected. The work also discusses in detail the widespread adoption of digital twin in personalized medicine, human health prediction, etc., as well as the drawbacks that accompany the advancement, such as data privacy issues, computing complexity, and the precision of human physiology simulation. The paper concludes that even though the twin technology is promising, it still needs major development in AI, big data, and ethics to become a reality and also questions the viability of completely achieving human digital twin. Another work^45^ provides a distinctive viewpoint on the evolution of digital twin in healthcare by highlighting the importance of incorporating ethical and social values in their architecture. The authors argue that, while there has been an extensive amount of work done towards the technological aspect of developing a digital twin model for a healthcare environment (monitoring, diagnosis, treatment of patient), there exists a need to explore the ethical and cultural implications of these technologies on human life. The study emphasizes on designing the healthcare digital twin model that carefully considers topics related to patient privacy, autonomy, equity, and openness in addition to addressing the technical difficulties. The work suggests finding a balance between innovation and responsible use of patient-related data by placing the creation of digital twin within larger social and ethical settings.

Some of the major gaps identified are that digital twin application and implementation especially in healthcare sector is still theoretical or in early implementation stages because real-time deployment presents certain barriers like, computational efficiency, scalability and integration of new technologies into existing healthcare infrastructure. Digital twins in healthcare rely on vast amounts of sensitive patient data, including genetic information, medical histories, lifestyle habits, and real-time health metrics from wearables. Continuous processing of such data and simulation in real time can lead to significant energy consumption, which may be a concern, especially for widespread use in healthcare settings with limited resources. Digital twin models, that evolve dynamically and use real-time data, require substantial computational power. Simulating the complex interactions of the human body, including genetic, physiological, and behavioural variables, demands high-performance computing infrastructure. As the number of digital twins grows, the computational resources required will also increase exponentially. Optimizing algorithms to scale effectively while managing the computational load remains an unresolved challenge. Many digital twin models in healthcare are still in early stages, and while they may show promising results in small-scale studies or simulations, they have not been fully validated across diverse patient populations or in real-world healthcare settings. There is a need for greater emphasis on the development of software and protocols that can bridge the gap between legacy healthcare systems and modern digital twin technologies. As mentioned earlier, digital twin uses patient sensitive data and the integration of such diverse data increases the risk of data breaches, unauthorized access, and misuse. In the context of real-time patient monitoring, the continuous nature of data collection (from sensors, wearables, and other sources) increases the risk of data being intercepted or mishandled. The review suggests that data privacy mechanisms may not be fully sufficient to protect sensitive health information, especially as real-time data from various sources accumulates. Ethical frameworks are insufficiently addressed in many of the studies. There is a strict need to consider the ethical implications of using such technologies, particularly concerning the autonomy of the patient and the handling of sensitive data.

## Problem statement

Healthcare industry is constantly evolving, driving the need for the integration of cutting-edge technologies to enhance patient care. Digital Twin technology is one such advancement that brings new revolution in healthcare by enhancing patient monitoring, disease prediction and even aiding in surgical procedures. As quoted earlier, a WHO report claims that Cardiovascular Disease (CVD) causes 17.9 million deaths each year, which accounts for about 31% of deaths globally. WHO also predicts that by 2030, heart disease will claim the lives of nearly 23.6 million people^11^. Due to poor food habits and sedentary lifestyle there has been a sharp increase in various heart disease cases. Heart is a complex and critical organ that requires continuous monitoring to detect various CVD. Current methods used to diagnose heart conditions such as cardiomegaly, arrhythmia and cardiomyopathy demand invasive procedures or infrequent checkups due to the infeasibility of visiting hospital often, thus leading to delayed diagnoses, which in turn delays preventive care. Another important point to note in case of cardiovascular diseases is that these conditions are reactive rather than proactive, meaning medical interventions happens only after the disease has significantly progressed.

Keeping the above points in mind, this paper proposes to use digital twin technology to revolutionize cardiovascular disease detection by creating a real-time, continuously updated model of patient’s heart using on-chest ultrasound (USG) sensor. This paper presents a framework for a digital twin enabled heart monitoring and prediction model, which includes layers that perform data collection, integration, analysis, prediction, and ensure data security. Using USG scans taken periodically over a set time, helps digital twin model to simulate and replicate the functional and anatomical properties of a patient’s heart, therefore offering a personalized patient health status and real-time monitoring capabilities to healthcare professionals.

Several challenges that were faced while designing the framework are; Data Quality Issues that arises while using X-ray images from USG sensor as input for the digital twin model, as the image should be of high quality to ensure accurate data interpretation since this is a non-invasive method for heart monitoring. Continuous monitoring requires exceptional computational power so that the data and computation accuracy are not compromised. Real-time data analysis, as well as predictive analysis also requires handling and analysis large volume of data. One of the biggest risks here is the data security. While the data is being transmitted to data storge point needs to be protected and secured in order to safeguard patient-sensitive information.

To overcome the above challenges and build a continuous monitoring and predictive digital twin model, the next section, proposes a “TwinCardio” framework, deigned to monitor patient’s cardiovascular health in real-time and also discusses how the data is acquired to how data is processed securely.

## Proposed digital twin framework for cardiac monitoring—TwinCardio

**Fig. 2:**
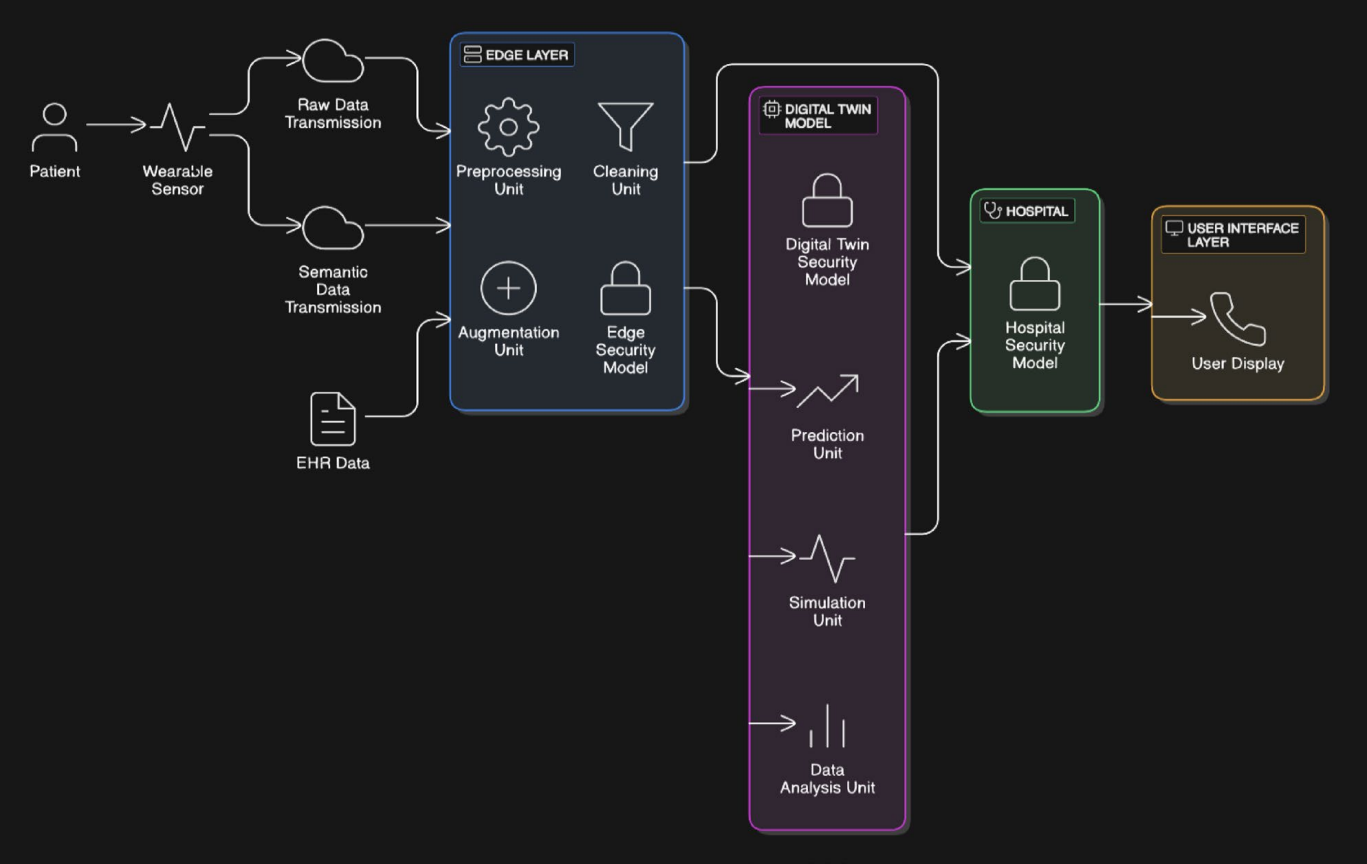
TwinCardio conceptual model.

An overview of the TwinCardio model has been described above in Fig. 2, focusing on the key entities, their relationships, process and scope of the model. The conceptual model in the figure, depicts a secure digital twin framework for patient monitoring using wearable sensors that captures ultrasound scan data. This sensor data along with EHRs is transmitted to edge layer for processing, cleaning and augmentation. The refined data from edge layer is fed to digital twin model, that performs prediction, simulation and analysis on patient data, protected by security protocols. Hospitals can access the model securely and the results will be displayed through a user interface to both clinicians and patients. This framework ensures, efficient, privacy-compliant monitoring system for personalized patient care.

**Fig. 3:**
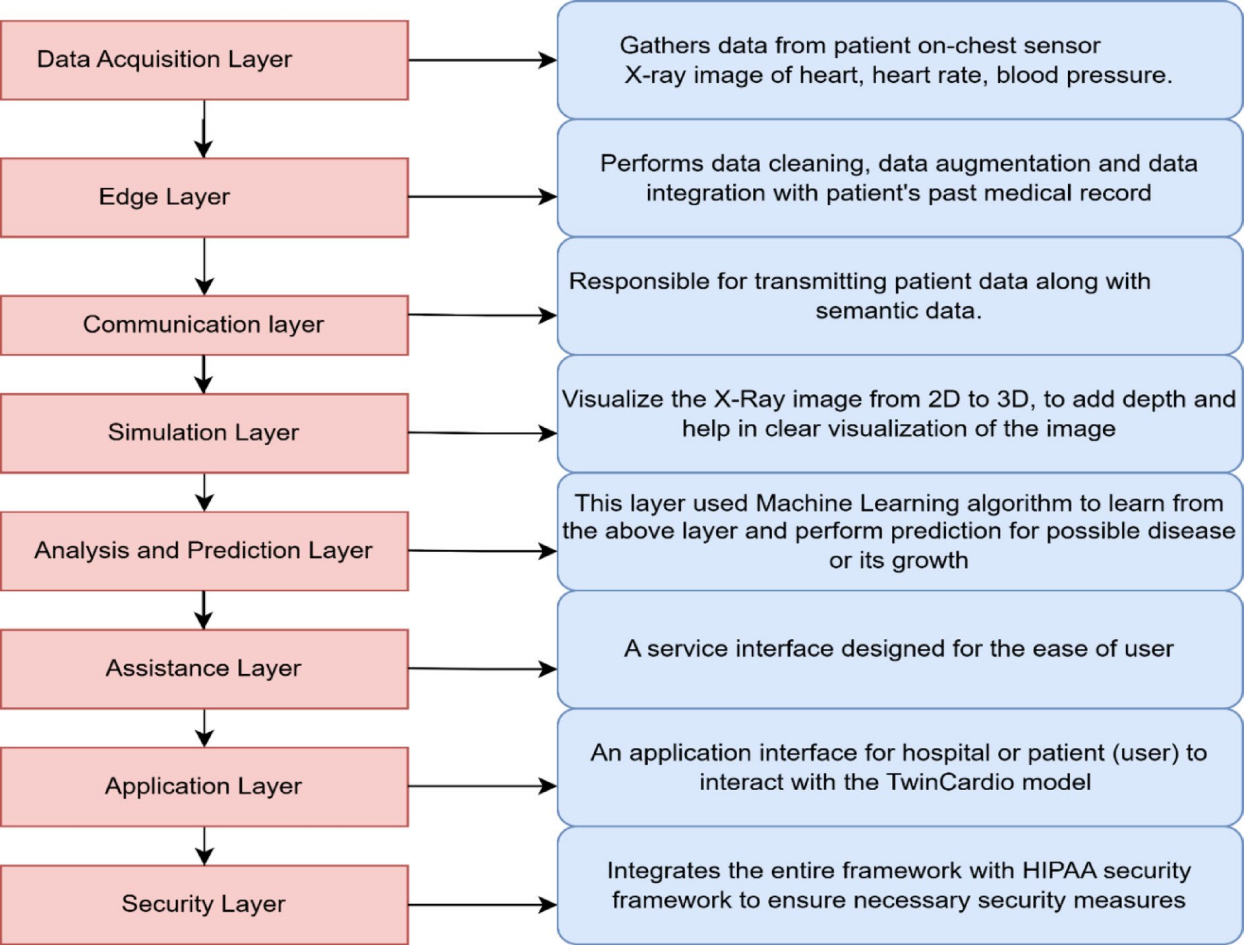
TwinCardio reference framework.

In this section, TwinCardio framework as seen in Fig. 3, proposes an 8-layer architecture for a Digital twin-enabled cardio monitoring model that defines the working methodology from data gathering to data representation to data interpretation. The proposed layers involve an IoT health sensor, Edge computation, and Digital Twin model for visualization and prediction of a patient’s heart health. TwinCardio aims to detect various abnormalities in heart of the patient: Cardiomyopathy, cardiomegaly and arrhythmia.

The reference framework is as follows.

### Data acquisition layer

There exists various scans to identify CVD, like EKG (Electrocardiography), echo (Echocardiography), CT scan, Cardio MRI etc. Among these, echocardiography is commonly used to examine the structural integrity and blood-delivery capabilities of the heart. It is an ultrasound of the heart that provides images of the heart’s chambers, valves, and blood flow. It can help diagnose conditions like heart failure, valve problems, and birth defects. TwinCardio proposes using a wearable echo sensor on the chest to scan ultrasound images of the heart. The implantation of the sensor device needs to ensure that the device is attached to the skin properly so that any friction caused by clothes or sweat doesn’t interfere with the sensor. The work^46^ presents a wearable device that performs continuous ultrasound scans of the heart to analyze and assess the latest condition of the heart and its functioning. The work also demonstrates the material fabrication of the device and how the device is attached to the skin for continuous and real-time monitoring of the left ventricle (the chamber of the heart) from different views.

**Fig. 4:**
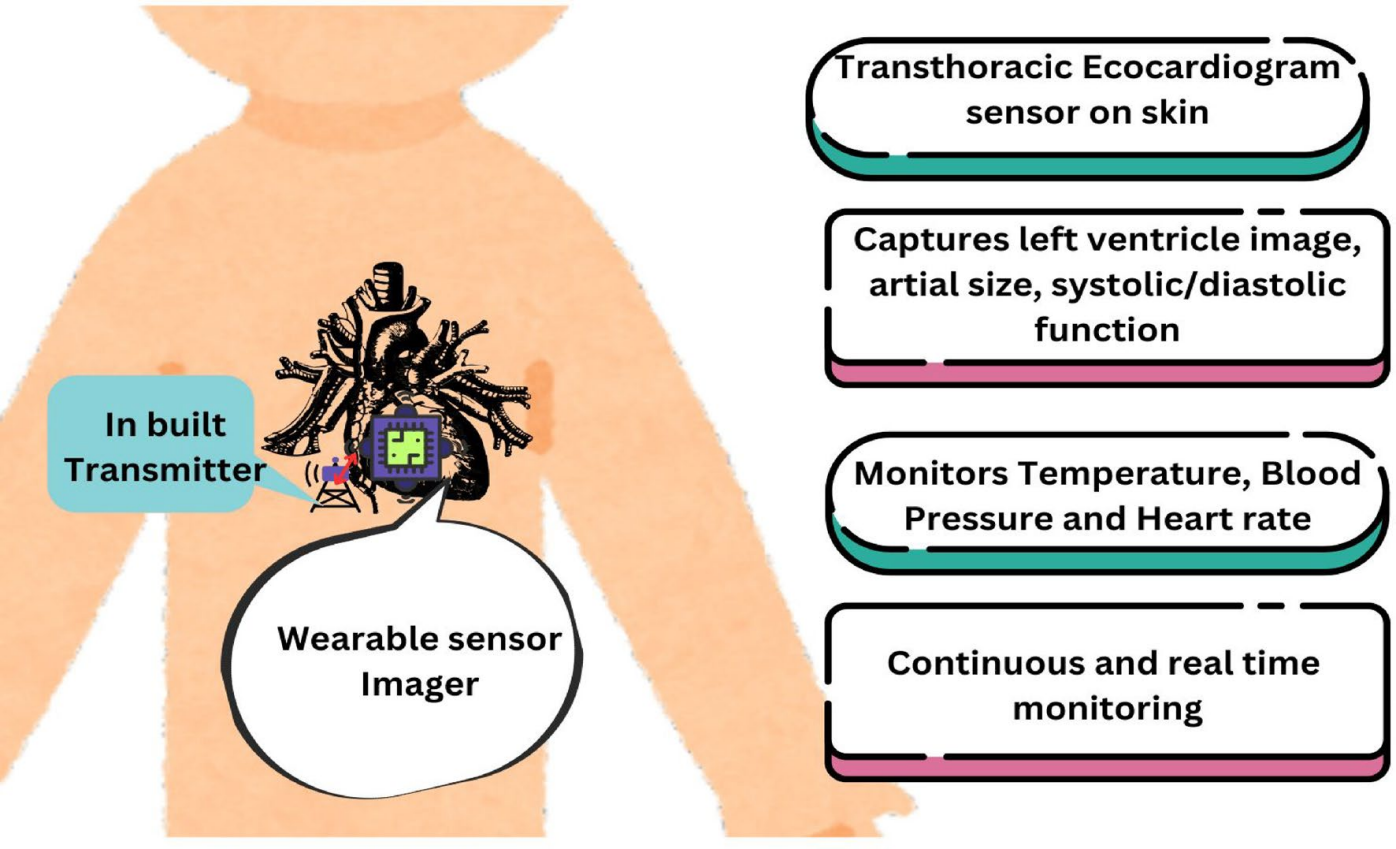
Outline of an on-skin wearable echo sensor^46^.

TwinCardio proposed work is to incorporate a transthoracic echocardiogram^46^ and capture an ultrasound scan of the heart by using continuous sound waves to generate images of the heart and its chambers. Figure 4 shows the on-chest sensor depiction along with the functionalities that will be performed by the sensor. The ultrasound scan is very helpful in evaluating the conditions of the left ventricle, right ventricle, size of the artery, size of the heart, and the heartbeat. This scan can help determining various cardiac conditions like cardiomegaly, the condition where the size of the heart increases more than normal; ischemia, where the blood supply to the heart is reduced; ventricle hypertrophy, a condition in which the lower chambers of heart thicken, making it difficult for the heart to pump blood; arrythmia, in this condition, the heart either beats too fast or too slow and has irregular rhythm, etc.^22^. These data serve as input from the physical entity and are used in the visualization and real-time monitoring of patients.

### Edge layer

Once the sensors capture the data, it is transmitted to the edge layer where the edge layer shares services that contribute towards data cleaning, data augmentation as well as integration with the patient’s medical history and serves as the bridge between sensor networks and conventional communication networks. In any medical record, a few attributes are mandatory to be present to identify the patient details, for example, the age, height, and weight of the patient also, another crucial attribute is the date, year and time when the data was collected, as these marks are the benchmark to compare past or future medical records. Data that contains the patient name, age, weight, height, etc., can be preloaded in the system to mark as patient identification. But the date and time stamp needs to be current and thus it has to be done in real-time. Therefore, TwinCardio adds the date and time stamp to the scan in the edge layer. This is done to reduce the load on sensors, and hence, any time-consuming task can be accomplished by the edge layer^47^. The sensor data is cleaned, pre-processed and then date and time stamp is added in edge layer. These data are then passed further for visualization, analysis and prediction.

Although edge layer has more processing power when compared to sensors, edge layer still suffers from issues like, limited storage capacity, sometimes important data may be overlooked in order to reduce latency, etc. To help with these drawbacks, as future work, the edge layer can be upgraded to collaborate with cloud computing, which address the limitations of the edge layer. Any computational task that needs be performed can be either done on edge layer or can be sent to cloud layer, if the processing power required is too high. In addition, all the data regardless of the importance can be store in the cloud and used later when required. This will lower the burden of edge layer as well provide a better and seamless experience to the parties (hospital, doctor, patient) involved in the system, therefore optimizing the information processing function^48^.

### Communication layer

TwinCardio is proposed to support bidirectional synchronization between the virtual twin of the heart and the patient to ensure a completely dedicated and real-time digital twin model. This communication between the device and the model involves information related to the heart size, heart beat and the left ventricle details, this makes the data highly time sensitive and apart from the cardiac data, another form of the data that will be transferred between the physical twin and the model will be the current environmental scenario, stress level, physical movements, etc. These data attributes that need to be transferred make it difficult for the current communication networks to manage them properly. Hence, there is a need to incorporate semantic data transfer, which paves way to Semantic Communication.^48^. Semantic communication focuses on the meaning interpreted by the receiver rather than the accuracy of the original data or signal^49^. This work proposes integrating raw sensor data transmission along with semantic communication to elevate the quality of data acquired and for better analysis of patient’s CVD health.

### Simulation layer

**Fig. 5:**
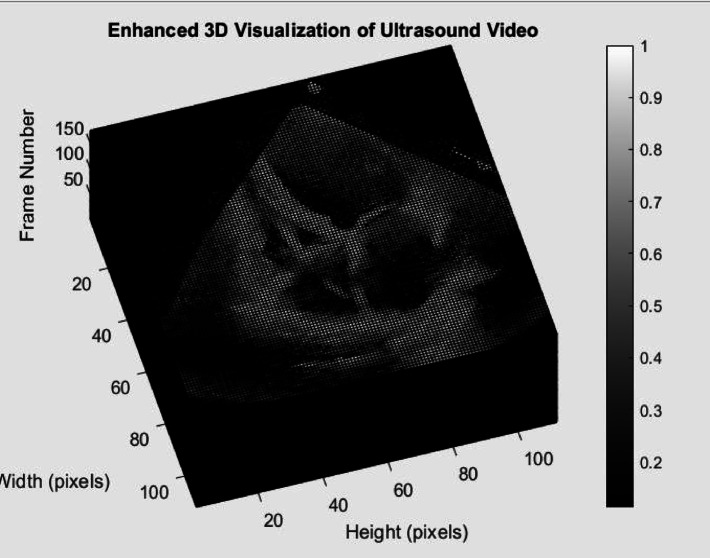
3D view of the ultrasound image of heart.

The ultrasound video is captured from the sensor, along with other attributes like heart rate, body temperature and blood oxygen level. The echo video, i.e. the ultrasound video is used to model a 3D image of the heart and other supporting data will be used further in the analysis phase. The software used for the simulation is MATLAB. The video is split into frames based on the video duration and frame rate and is used to sculpt the 3D model, depicted in Fig. 5. The echo data is taken and visualized to allow the heart to be seen from different angle but within the range of the ultrasound image. For this study and 3D visualization demonstration, the echo dataset has been taken from Stanford University—“Echo-Net Dynamic” dataset. The dataset contains 10,030 echo videos that can be used to train the model further.

### Analysis and prediction

Once a 3D model is created, TwinCardio proposes to apply deep reinforcement learning (DRL) algorithm (future work) to annotate the model, i.e. generate labels for the heart image and then incorporates the additional information that was gathered, like heart rate, EKG values, blood oxygen level and semantic information form the edge layer and create a monitoring dashboard. Deep reinforcement learning can be trained to identify the anomalies in the image, which are highlighted on the 3D model.

DRL can also be used to predict the condition of cardiovascular health by taking the following data into consideration, stress level, heart rate, blood oxygen level. Elevation or deduction in any of the levels serve as marker for prediction as well as comparison based on patient’s medical history.

### Assistance layer

This layer provides a service interface to better aid the user by providing different services, which will include request of a service, its response and additional information about patient medical records. These interfaces provide interactive support for service provision, service request and platform operation. The service request interface provides functions or updates to healthcare institutions by providing patient records and current monitoring data while simultaneously providing patients with the necessary interface to access the treatment plans and raise queries to healthcare organisations. Thus, it enables human and machine interaction for remote patient treatment and diagnosis^50^.

### Application layer

This layer allows the user (hospital or patient) to interact with the TwinCardio platform. The designed application will build a bridge between the hospital and the patient by providing services like—remote consultation, report updation, real-time monitoring data from sensors, alarms to detect and notify anomalies, and so on. This layer will have a different front-end access for patient and a different one for the hospitals. This can be imagined as a separate login for admin and a user. Both patient and the clinicians can access TwinCardio, but the functionalities provided to them will differ. For a patient—all the basic patient information, along with their EMRs, scan reports will be available. Patients can visualize their vital statistics, receive alerts, and track the effects of lifestyle changes or medications. The twin can serve as an educational interface, helping patients understand their condition and collaborate with clinicians on treatment plans. Whereas, for the hospital, the above-mentioned patient data will be visible to doctors, along with the option to monitor the simulation model, add different features for monitoring, drop irrelevant features etc., will be made available. Doctors can further use the digital twin to simulate disease progression, assess treatment outcomes, and tailor personalized therapies. They may even access real-time data for routine monitoring^29^.

### Security layer

TwinCardio framework combines various technologies like IoT, edge computing, Digital Twin, communication networks, etc., and opens new concerns in terms of security and privacy. An attacker able to penetrate model and has the potential and capability to harm or terminate the life of the patient by reprogramming devices. There are three types of targets when attacking these systems^51^:

Patient: The attacker can target directly to the patient’s health. It means attacking to the sensing, processing communication aspects of the TwinCardio.

Data: An attacker can access highly confidential and sensitive data belonging to the patient or the involved ones in the medical treatment. The loss of data privacy can lead to potential blackmailing, computer abuse and discrimination.

Device: An attacker can perform a Denial of Service (DoS) attack on the application layer, or also be part of it (e.g., wearable or implanted device), and deploy it to belong to a huge botnet in order to perform robust Distributed Denial of Service (DDoS) attacks. Moreover, this can also result in privacy loss over systems that should be designed to fail open as suggested.

To deal with numerous security, privacy and confidentiality threat, the work aligns itself with HIPAA rules^52^. The Health Insurance Portability and Accountability Act (HIPAA) is a U.S. law designed to protect patient privacy and ensure the security of healthcare information. When applied to healthcare digital twin models, HIPAA provides a framework for managing sensitive health data while ensuring compliance with privacy and security regulations. HIPAA prescribe certain mandatory rules like, Protected Health Information (PHI), Minimum necessary rules, Data Integrity and Quality assurance etc., that are used to monitor and regulate healthcare digital twin models.

Under HIPAA, Protected Health Information (PHI) refers to any data that identifies an individual and relates to their health status, care, or payment for care. In terms of healthcare digital twin, PHIs include data that are collected from wearable devices on patients, current and past electronic health records (EHRs) of the patient, genetic data, patient DNA profile or dental record, etc., and other patient information. HIPAA acts as a central security body that ensures all patient-related data remains strictly confidential as well and any use or access of the data should have explicit consent from the patient. In case of healthcare digital twin model, patient consent has to be taken prior to collecting, analysing as well as sharing the data. Also, the patient must be informed beforehand regarding how their data will be used in a digital twin model and what will be the safeguards used to protect the sensitive information.

According to the minimum necessary rule described by HIPAA, only the minimum required PHI that can be used to achieve the intended purpose, such as precise modeling and medical data analysis, should be accessed and shared by the digital twin model. HIPAA is required to protect as well as manage patient EHRs by applying administrative protections, which entails setting up policies in place for incident response, data access and handling, employee training, etc. These rules apply to digital twin models when handling patient-sensitive information to guarantee authorized as well as limited access and view of health data^53^.

Ensuring that only authorized individuals have access to the digital twin data requires the implementation of robust access control methods, like user authentication and permission systems. HIPAA ensures that all the EHRs that are being transmitted and stored by the model must be accurate and unaltered, which can be achieved by implementing controls (validation process and regular checks) that monitor the integrity and correctness of the information that is being used for visualizations and analysis by the digital twin model. Data correctness and integrity are checked from the source of data being collected throughout the use and storage of data as well. Medical data are very sensitive data and are to be handled with a lot of care.

## Experimental setup

Now that the proposed framework is established for collection, processing and simulation of the ultrasound scan data, this paper targets another important task of cardiovascular disease analysis, i.e. classification and prediction based on Electronic Medical Records. This section discusses the dataset used in the paper as well as the model designed for disease classification in detail.

### Dataset description

From healthcare point of view; there exist multimodal data sources such as: structured data (Lab test results, vital signs, demographics), unstructured data (Clinical notes, discharge summaries, pathology reports), medical images (X-ray, ultrasound, CT scan), genomic data (DNA, gene expression), time-series data (data captured from sensors), etc. For cardiovascular disease prediction and monitoring using Digital Twin, the proposed work considers following data sources: Electronic Medical Records (structured data), ultrasound scan videos (medial image data) and wearable sensor data (time-series). EMRs are utilized as the primary data source for disease prediction and classification. EMRs offers a deeper essence of the patient health, which will help predict and monitor future changes in their cardiovascular health. EMRs are structured records that contain historical and clinical variables pertinent to a patient’s cardiovascular health, making them highly suitable for training supervised machine learning models^54^. Another data format incorporated in TwinCardio model are ultrasound scans, based on these scan data a simulation model is created which will be an extension of current work that will be used to classify and predict based on 3D heart model. This paper, however, focuses exclusively on the analysis and modelling of EMR data for the classification of cardiovascular disease and prediction of the disease based on the data features present in the medical record.

Cleavland Heart Disease Dataset^55^ has been used to train the proposed model—TwinNet, as well as test existing regression and classification methods. This dataset consists of 303 patient records and includes a comprehensive set of 13 clinical features: ‘gender’, ‘chest_pain_type’, ‘max_heart_rate_achieved’, ‘fasting_blood_sugar’, ‘resting_electrocardiogram’, ‘resting_blood_pressure’, ‘exercise_induced_angina’, ‘st_slope’, ‘st_depression’, ‘thalassemia’, ‘age’, ‘num_major_vessels’, and ‘cholesterol’, and one target value. Each of these features has a potential role in influencing the onset or presence of cardiovascular disease. The dataset undergoes a thorough preprocessing phase, which includes handling missing values, encoding categorical variables, and normalization of numerical features. This preprocessing ensures data consistency and quality for downstream analysis. Understanding the distribution of each feature is a fundamental aspect of the experimental design. Analysing feature distribution provides insight into the variability and statistical properties of the data, such as skewness, modality, and potential outliers. These factors can significantly influence model performance. In particular, it allows the identification of features that may disproportionately affect the learning process or bias predictions.

### Dataset summary

Data size: 303 rows and 14 columns (13 independent + one target variable) Dataset has no missing values. Six features in the dataset are numerical and the rest (seven features) are categorical variables. Target variable is fairly balanced, 54% no-disease to 46% has-disease. After performing various correlations, it can be concluded that the numerical features ‘num_major_vessels’, ‘max_heart_rate_achieved’ and ‘st_depression’ are reasonably fairly correlated with the target variable at − 0.47, 0.43 and − 0.43 correlation coefficient respectively. The categorical features ‘chest_pain_type’, ‘num_major_vessels’, ‘thalassemia’, and ‘exercise_induced_angina’ are better correlated with the target variable, ‘thalassemia’ being the highest at 0.52. Surprisingly, according to the dataset, ‘cholestrol’ has less correlation with heart disease. Features that have higher predictive power could be, ‘chest_pain_type’, ‘num_major_vessels’, ‘thalassemia’, ‘exercise_induced_angina’, ‘max_heart_rate_achieved’ and ‘st_depression’.

### Exploratory data analysis

Before initiating any modelling steps, the dataset undergoes a comprehensive exploratory data analysis (EDA) phase to understand the nature and impact of each feature on cardiovascular disease prediction. To examine how the distribution of features varies across patients with and without cardiovascular disease, several plots are generated and analyzed:

#### Kernel density estimate (KDE) plots for numerical features

**Fig. 6:**
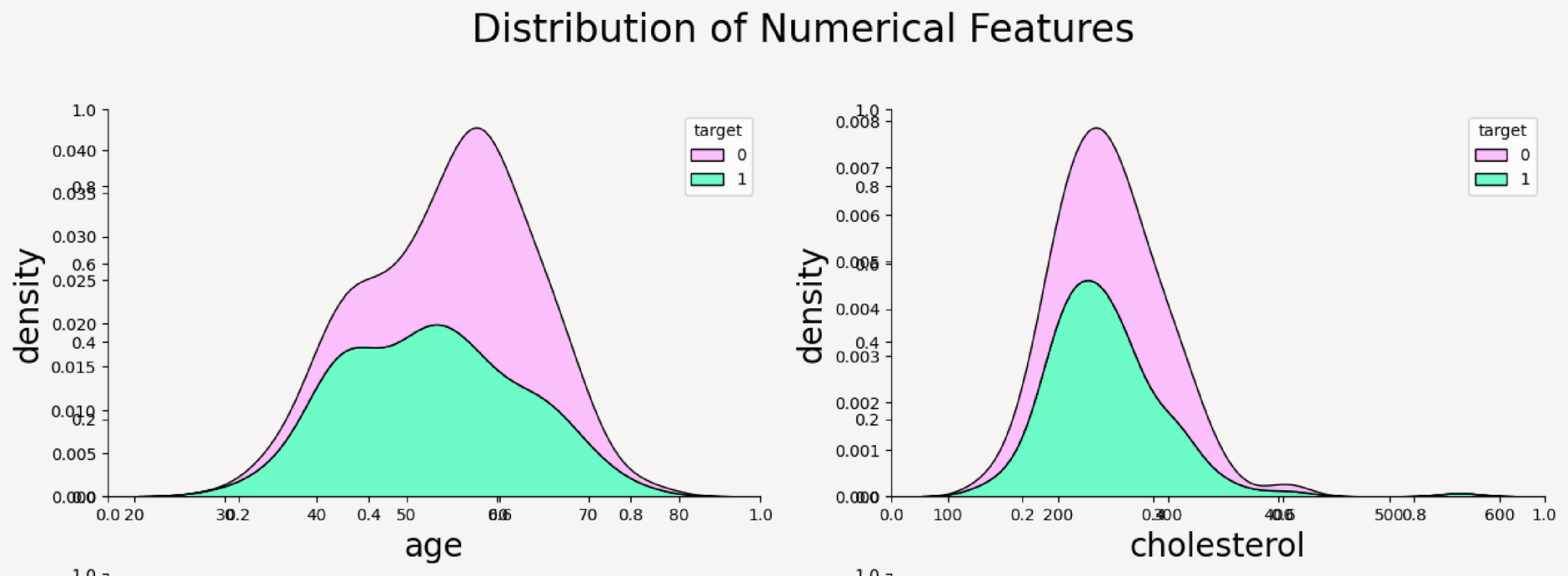
(**a**) Density distribution of numerical feature age. (**b**) Density distribution of cholesterol.

**Fig. 7:**
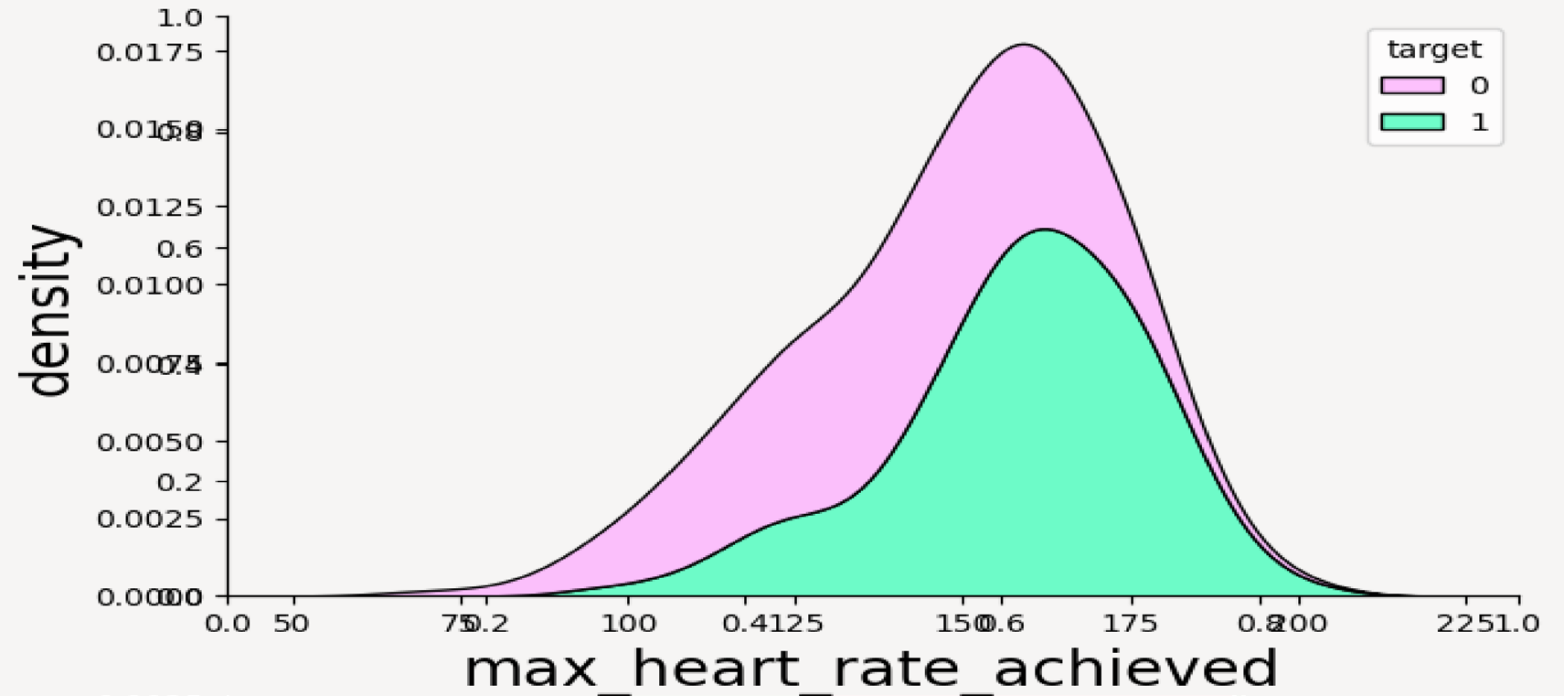
Density distribution of numerical feature max_heart_rate_achieved.

**Fig. 8:**
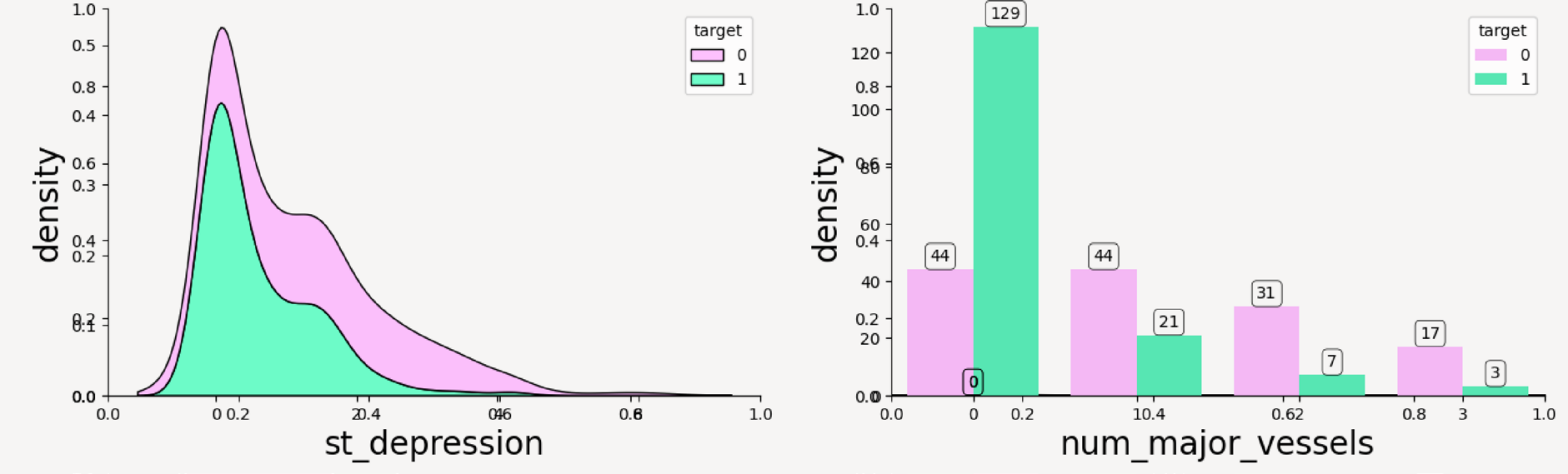
(**a**) Density distribution of st_depression. (**b**) Density distribution of num_major_vessels.

KDE plots are employed for all numerical features using seaborn’s kdeplot function^56^. These plots reveal the shape, spread, and skewness of the data and how they differ across target classes (target = 0 for no disease, target = 1 for disease). These plots help assess which features contribute meaningfully to target prediction by comparing their probability distributions across classes. In general, the greater the divergence between the distributions for target = 0 and target = 1, the more informative the feature is likely to be. For instance:

From the above plots it can be inferred that the numerical features have following impact on the outcome of CVD—such as; age: Both target groups are normally distributed and it can be noticed that people with heart disease (target = 1) tend to be slightly older on average than those without heart disease. Cholesterol: Cholesterol levels are right-skewed for both groups and the target = 0 group shows higher density in the 200–300 range. There’s considerable overlap, suggesting cholesterol alone may not be a strong discriminator as shown in Fig. 6a, b. max_heart_rate_achieved: This is a very informative attribute and clearly shows that individuals without heart disease reach higher maximum heart rates. There’s a noticeable separation, where target = 1 group has a left-skewed distribution, meaning lower max heart rates. This variable might be a strong predictor as shown in Fig. 7. st_depression: Strong difference—people with heart disease have higher st_depression (ECG abnormality after exercise). The target = 0 group peaks at near-zero values, while target = 1 has a wider spread toward higher values thus proving to be a very good feature for classification. num_major_vessels: People without heart disease tend to have more major vessels colored by fluoroscopy (0–3). Target = 1 group has more zeros, indicating fewer visible vessels—possibly due to blockage or imaging results. Strong separation suggests high predictive value as shown in Fig. 8a, b.

#### Count plots for categorical features

Count plots are used to visualize the distribution of categorical variables across the two target classes^57^. These are particularly useful for understanding imbalances or skew in category occurrences. These count plots not only reveal which categories dominate in each class but also guide feature encoding choices (e.g., one-hot encoding vs. label encoding) based on the number of categories and their predictive relevance.

**Fig. 9:**
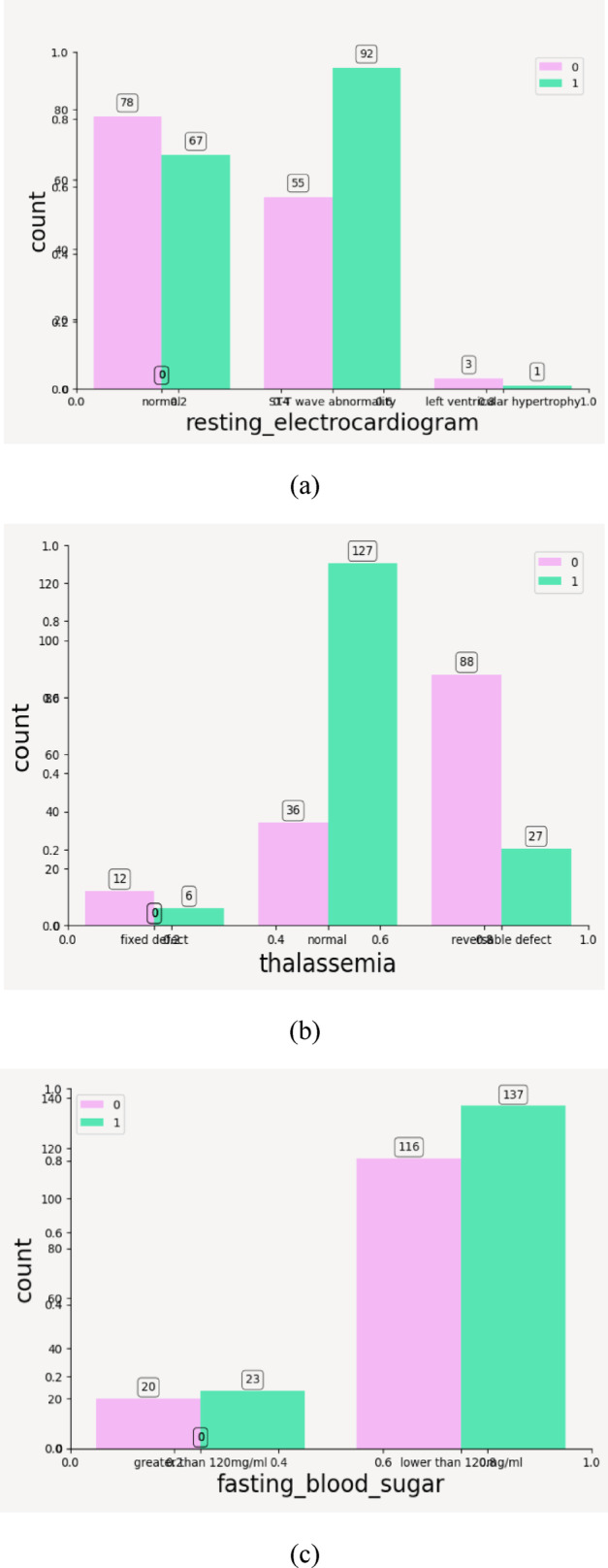
(**a**) Count plot for categorical values resting_echo. (**b**) Count plot for Thalassemia. (**c**) Count plot for fasting_blood_sugar.

**Fig. 10:**
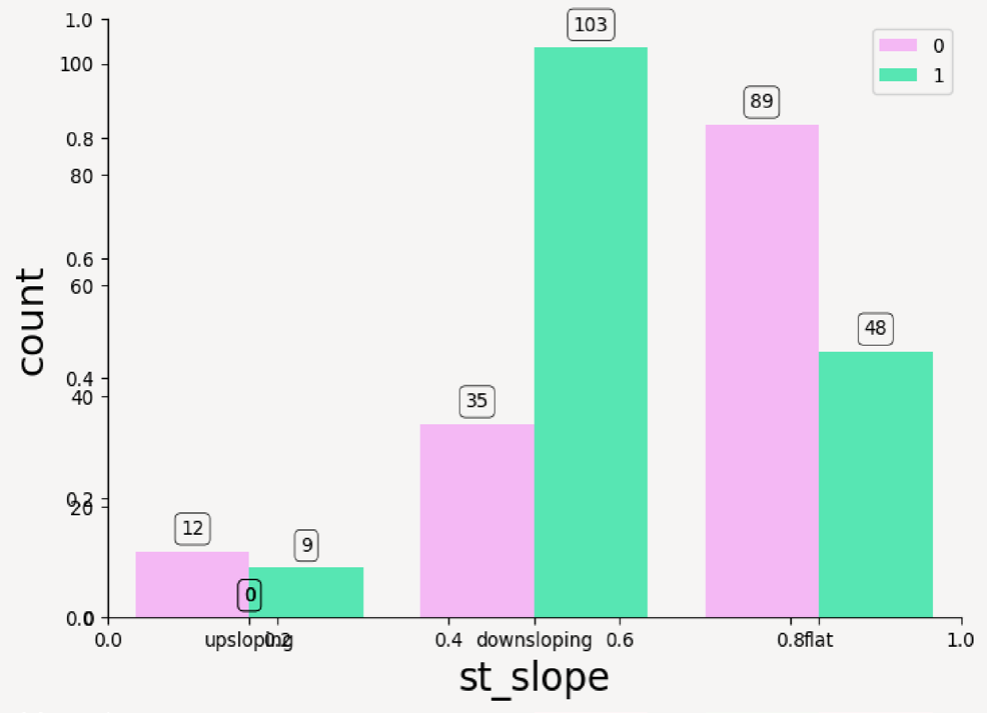
Count plot for categorical value st_slope.

**Fig. 11:**
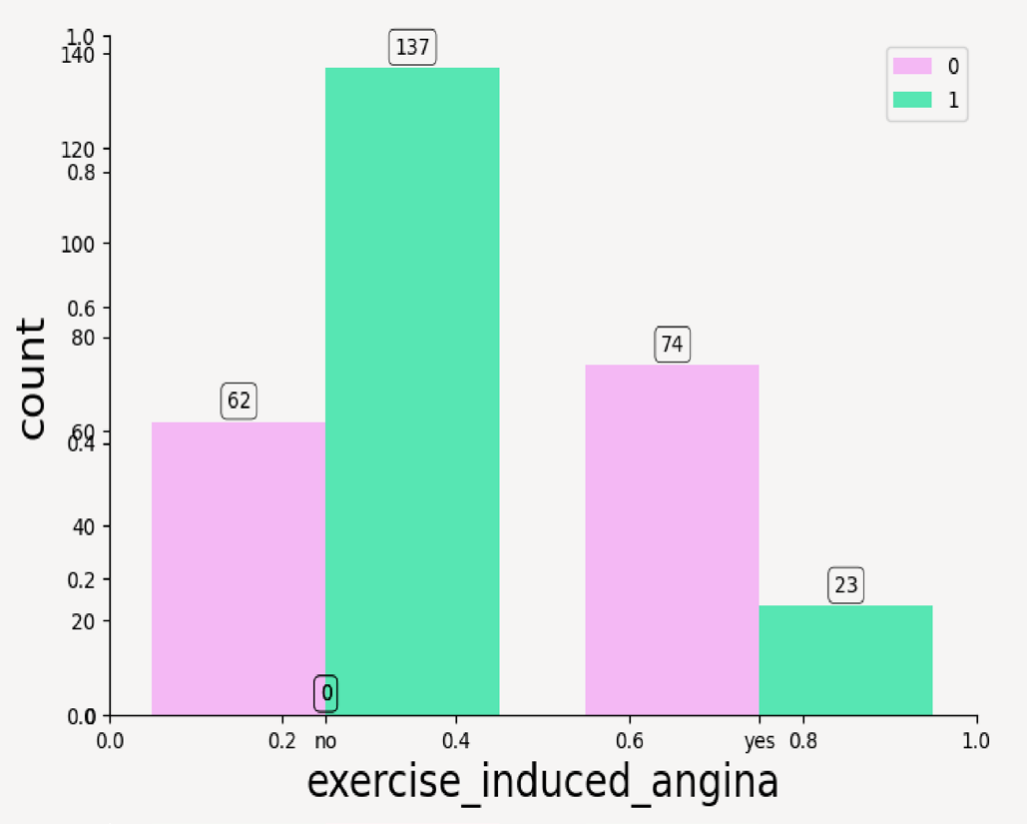
Count plot for categorical value exercise_induced_angina.

From above plots it can be inferred that the categorical features have following impact on the outcome of CVD—such as; Resting Electrocardiogram: ST-T wave abnormality (label 1) in Fig. 9a is more frequent in target 1 and left ventricular hypertrophy (label 2) is rare but slightly more in disease cases, thus it can be concluded that abnormal ECG results are predictive of disease. Thalassemia: In Fig. 9b, it can be seen that reversible defect (label 3) is more common in target 1 while normal thalassemia is found more in healthy individuals, thus suggesting a strong relationship between thalassemia type and disease. Fasting Blood Sugar: Majority of individuals have sugar < 120 mg/dl (label 0), there is not much distinction between classes as seen in Fig. 9c—this feature may not be highly predictive. ST Slope: Flat (label 2) ST slope dominates in heart disease cases with upsloping is mostly seen in healthy individuals (target 0) and downsloping appears somewhat balanced between both targets. A flat ST slope could be a red flag for heart disease.as shown in Fig. 10. Exercise-Induced Angina: Absence of angina (no, label 0) is much more common in the disease class whereas presence of angina (yes, label 1) appears more often in the healthy class. This is counterintuitive and may reflect other underlying correlations as shown in Fig. 11.

### Modelling

Following data preprocessing and exploratory analysis, the modeling phase proceeds through a structured and progressive approach. This involves first establishing a baseline using the Light Gradient Boosting Machine (LightGBM), followed by designing and optimizing a custom deep neural network *TwinNet*, and finally developing a stacked ensemble model that integrates both.

#### Light gradient boosting machine (LightGBM)

This algorithm is a tree-based gradient boosting framework that builds additive models in a forward stage-wise fashion^58^. At each iteration, it fits a decision tree to the negative gradient of the loss function with respect to the model’s output. The model minimizes the loss function by adding weak learners sequentially:

1 $${\varvec{F}}_{{\varvec{m}}} \left( {\varvec{x}} \right) = \user2{ F}_{{{\varvec{m}} - 1}} \left( {\varvec{x}} \right) + {\varvec{\gamma}}_{{\varvec{m}}} {\varvec{h}}_{{\varvec{m}}} \left( {\varvec{x}} \right)$$

- ${\varvec{F}}_{{\varvec{m}}} \left( {\varvec{x}} \right)$ is the prediction of the ensemble after ${\varvec{m}}$ trees,
- ${\varvec{h}}_{{\varvec{m}}} \left( {\varvec{x}} \right)$ is the new decision tree trained at iteration ${\varvec{m}}$,
- ${\varvec{\gamma}}_{{\varvec{m}}}$ is the learning rate or step size.

In classification, typically the **binary cross-entropy loss** is used:

2 $$L\left( {y,\hat{y}} \right) = - \left[ {ylog\left( {\hat{y}} \right) + \left( {1 - y} \right)log\left( {1 - \hat{y}} \right)} \right]$$

where $y \in \left\{ {0,1} \right\}$ is the true label, and $y \wedge \in \left[ {0,1} \right]$ is the predicted probability.

This performance demonstrates that the model generalizes well on the data, but still leaves room for improvement in sensitivity and robustness, particularly on small datasets where overfitting is a matter of concern.

#### TwinNet—custom neural network model

**Fig. 12:**
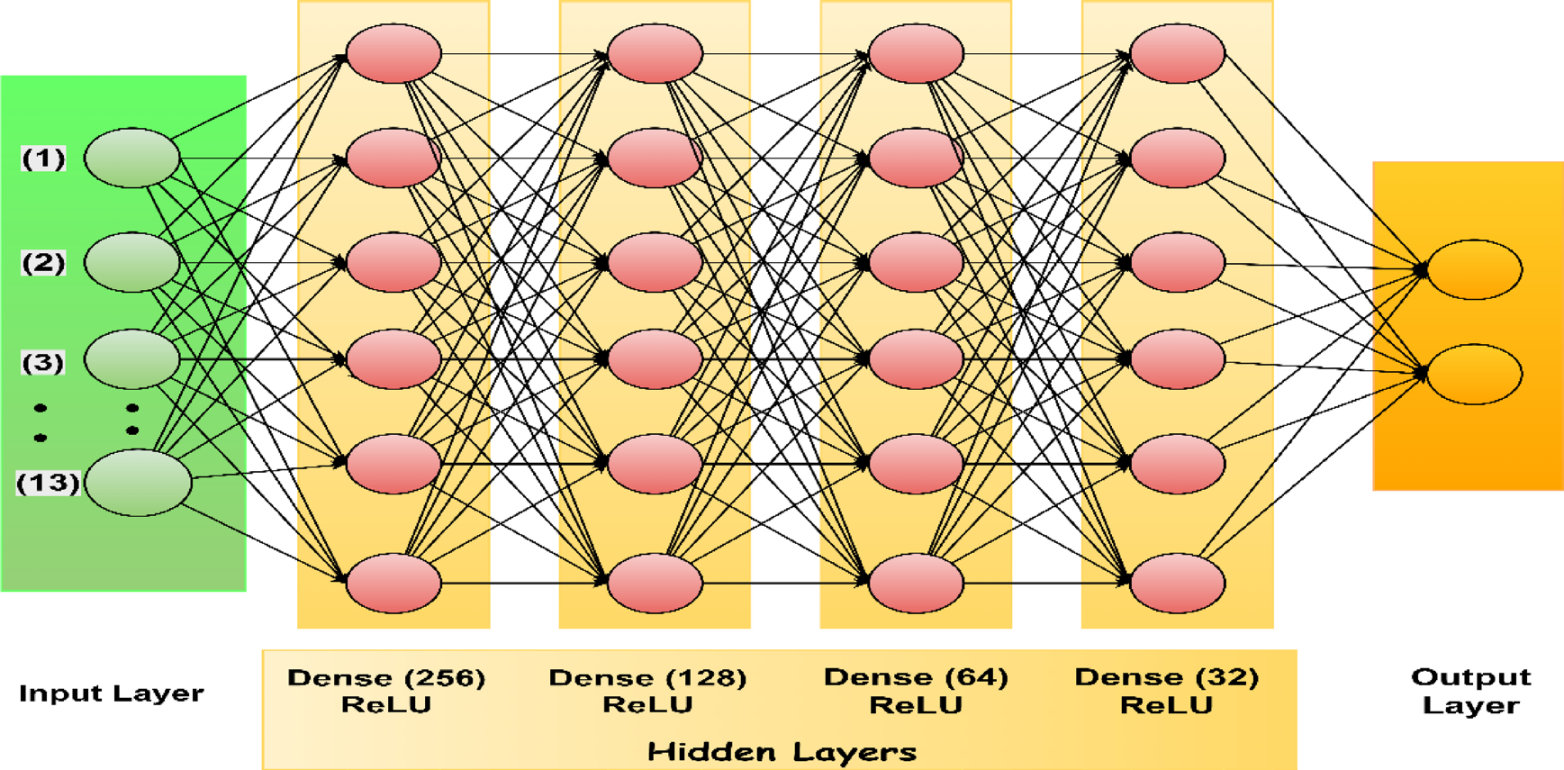
TwinNet: proposed fully connected feed-forward neural network for CVD classification.

**Fig. 13:**
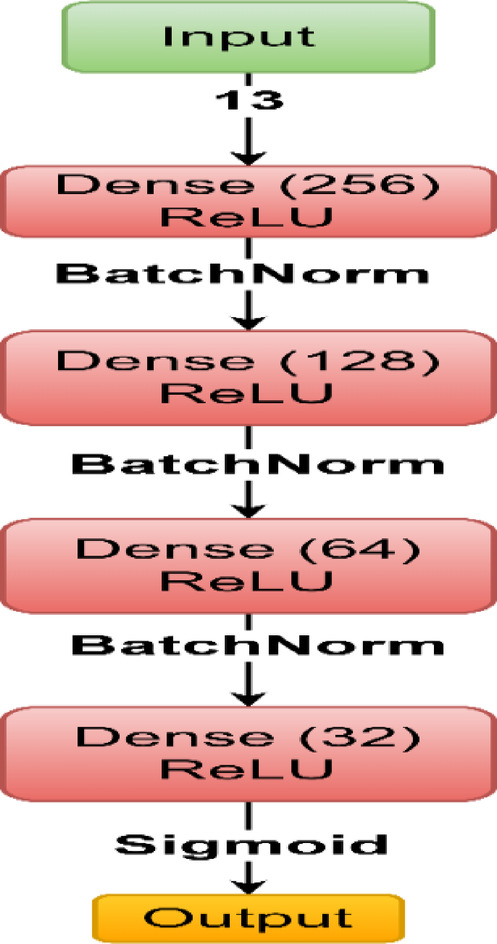
Flowchart of TwinNet parameters.

To overcome the limitations of LightGBM and capture more complex, non-linear patterns inherent in medical data, the proposed work introduces a **custom-designed fully connected feed-forward neural network—TwinNet** represented in Fig. 12. The architecture is developed through multiple layers and hyperparameter optimization as shown in Fig. 13 as well as in Table 2.

**Table 2:** Model parameter description.

Parameter	Values
Input layer	13 input features
Hidden layers	Four fully connected layers with 256, 128, 64 and 32 neurons respectively
Activation function	ReLU
Output layer	Single neuron with sigmoid activation for binary classification
Loss function	Binary cross-entropy
Optimizer	Adam optimizer
Learning rate	0.001
Training epochs	50
Batch size	64

TwinNet was trained using LeakyReLU, Swish and ReLU activation function, out of which ReLU performed best, this was decided when the validation accuracy dropped for above mentioned activation function except for ReLU. As well as, Adam optimizer and AdamW optimizer were also used, out of which Adam optimizer performed better. The drawback while using the other activation functions as well as optimizers was that, the validation accuracy either dropped or stayed stagnant as well as overfitting of results were also noticed in those cases. Apart from this, hyperparameter tuning was done which resulted in the above mentioned hyperparameters yielding best results.

The forward propagation is represented as:

3 $${\varvec{z}}^{\left( 1 \right)} = {\varvec{ReLU}}\left( {{\varvec{W}}^{\left( 1 \right)} {\varvec{x}} + {\varvec{b}}^{\left( 1 \right)} } \right)$$

4 $${\varvec{z}}^{\left( 2 \right)} = {\varvec{ReLU}}\left( {{\varvec{W}}^{\left( 2 \right)} {\varvec{z}}^{\left( 1 \right)} + {\varvec{b}}^{\left( 2 \right)} } \right)$$

5 $${\varvec{z}}^{\left( 3 \right)} = {\varvec{ReLU}}\left( {{\varvec{W}}^{\left( 3 \right)} {\varvec{z}}^{\left( 2 \right)} + {\varvec{b}}^{\left( 3 \right)} } \right)$$

6 $$\hat{\user2{y}} = {\varvec{\sigma}}\left( {{\varvec{W}}^{\left( 4 \right)} {\varvec{z}}^{\left( 3 \right)} + {\varvec{b}}\left( 4 \right)} \right)$$

The network is trained using binary cross-entropy:

7 $$L_{TN} = - \frac{1}{N}\mathop \sum \limits_{i = 1}^{N} \left[ {y_{i} \log \left( {\widehat{{y_{i} }}} \right) + \left( {1 - y_{i} } \right)\log \left( {1 - \widehat{{y_{i} }}} \right)} \right]$$

The model is trained using early stopping to avoid overfitting and evaluate performance based on the validation set. This marks a clear improvement over the LightGBM model, particularly in recall and ROC AUC, indicating better identification of true positive cases (patients at risk).

#### Stacked ensemble model

**Fig. 14:**
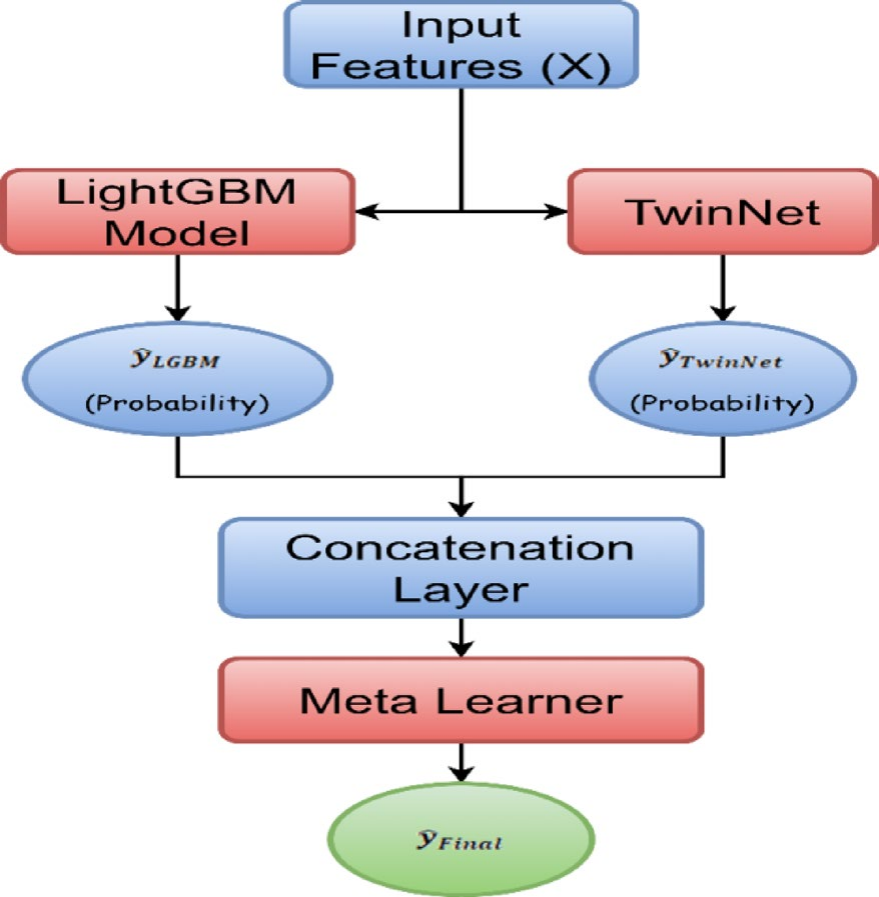
Stacked ensemble model—LGBM + TwinNet.

To further improve predictive performance, the study constructs a stacked ensemble that combines predictions from the LightGBM and neural network models as depicted in Fig. 14. The ensemble is implemented using logistic regression as a meta-learner over the base model outputs. Let:

- $\hat{y}_{LGBM}$ be the output from the LightGBM,
- $\hat{y}_{NN}$ be the output from the neural network.

The ensemble prediction is computed using a logistic regression meta-model:

8 $$\hat{y}_{Ensemble} = \sigma \left( {w_{1} \hat{y}_{LGBM} + w_{2} \hat{y}_{NN} + b} \right)$$

where $w_{1}$, $w_{2}$ and $b$ are learned parameters that balance the contribution of each base model.

This logistic regression-based stacking is trained on the out-of-fold predictions from both base models and validated on the hold-out test set.

The ensemble marginally improves the overall performance, particularly in balancing precision and recall, but does not outperform the custom neural network significantly in ROC AUC.

#### Model performance

Based on the above results which are consolidated in Table 3, TwinNet outperforms both the LightGBM and ensemble model across most key metrics. Its higher recall and ROC AUC suggest it is more effective at identifying patients with cardiovascular disease without increasing false positives significantly. Therefore, the study concludes that the neural network model is best suited for deployment in the current system.

**Table 3:** Designed and tested model performance metrics (in percentage).

Models	Accuracy	Precision	Recall	F1-score	ROC_AUC
LightGBM	88	89	88	88	89
TwinNet	93.2	89	92	90	99
Stacked Ensemble	91	92	86	89	98

Accordingly, the paper proceeds with the custom neural network model, TwinNet as the final model for cardiovascular disease prediction.

**Fig. 15:**
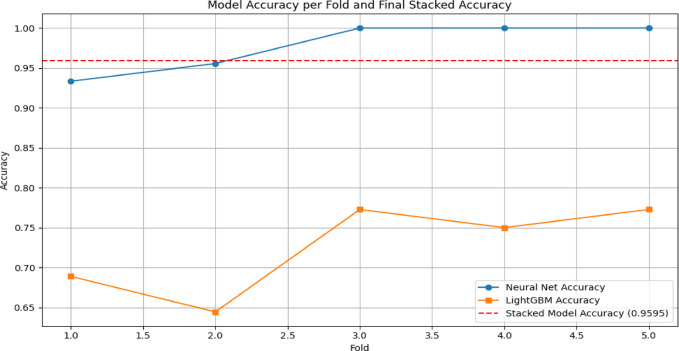
Model Accuracy comparison of all three model.

The graph in Fig. 15 illustrates the accuracy of two distinct models, TwinNet and LightGBM, across five folds, along with the final stacked model accuracy. The x-axis represents the fold number, ranging from 1 to 5, while the y-axis signifies accuracy, ranging from 0.60 to 1.00. The blue line with circular markers indicates the accuracy of the TwinNet model, which starts at approximately 0.93 in the first fold and steadily improves, reaching 1.00 by the fifth fold. Conversely, the orange line with square markers represents the LightGBM accuracy, which starts at about 0.70 in the first fold, dips to nearly 0.63 in the second fold, and then fluctuates between 0.65 and 0.70 in the subsequent folds. A red dashed line marks the final stacked model accuracy at 0.9595, showcasing the advantage of combining multiple models to achieve better predictive performance. The contrast in accuracy trends between the TwinNet and LightGBM highlights differences in their behavior and strengths across various folds. Overall, this graph underscores the effectiveness of model stacking in improving accuracy, making it a valuable approach in machine learning applications where precision is crucial, such as medical diagnostics.

## Result and discussion

This section discusses different performance metrics used to measure the strength of all the three models.

### Performance metrics

In machine learning model evaluation, especially for classification tasks, several diagnostic plots are used to understand different aspects of model performance^59,60^.

The **Calibration Curve** (also called the reliability curve) assesses how well predicted probabilities align with actual outcomes. It plots predicted probabilities against the actual frequency of positive outcomes. Ideally, a well-calibrated model will produce a diagonal line, meaning that for predictions with 0.7 confidence, the true proportion of positives is also 0.7. The formula for calibration in each bin is:

9 $$Calibration\;Error = \left| {\frac{1}{n}\mathop \sum \limits_{i = 1}^{n} \hat{p}_{i} - \frac{True\;Positives\;in\;binntives\;in\;bin}{n}} \right|$$

where $\hat{p}_{i}$ are the predicted probabilities in the bin.

**Fig. 16:**
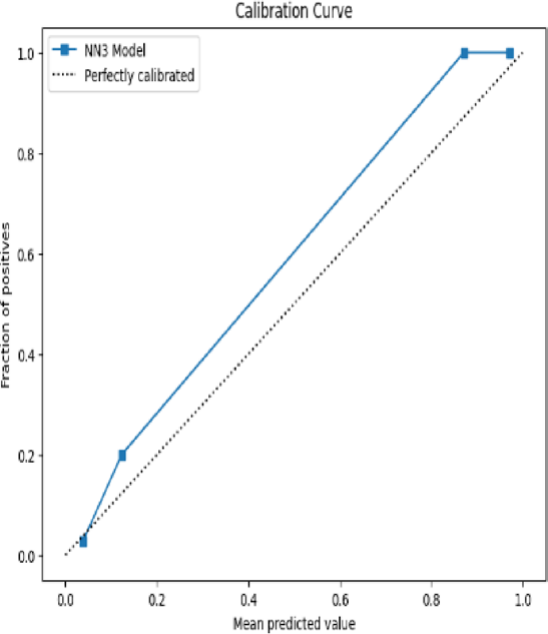
Calibrated curve for TwinNet model.

**Fig. 17:**
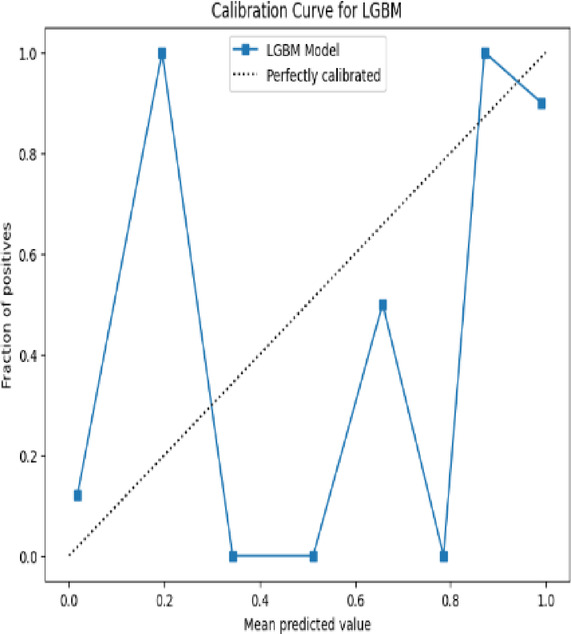
Calibrated curve for LGBM model.

**Fig. 18:**
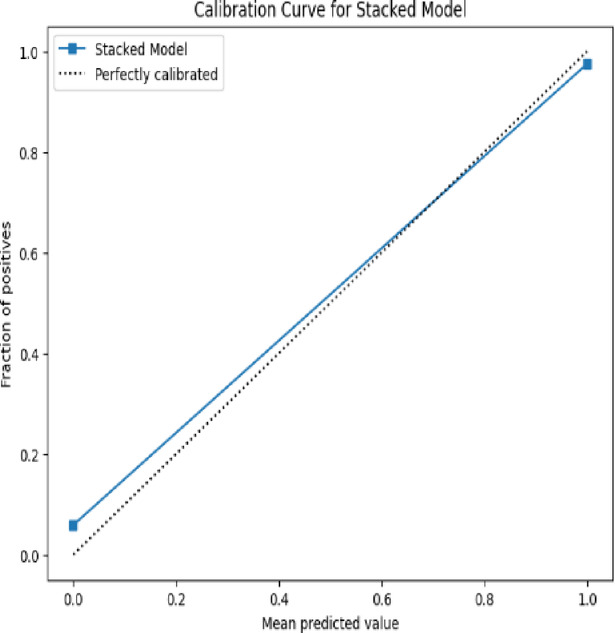
Calibrated curve for Stacked model.

The calibration curve for TwinNet in Fig. 16, for LGBM model in Fig. 17 and for stacked model in Fig. 18, are shown below;

Figure 16 shows the **calibration curve**, which evaluates how well the predicted probabilities align with actual class outcomes. In this plot, the blue line represents the predictions of the NN3 model, while the dotted black line corresponds to perfect calibration, where predicted probability exactly matches the true likelihood of a positive class. The proximity of the NN3 model’s calibration line to the ideal diagonal indicates that the model is well-calibrated—its predicted probabilities are realistic and trustworthy. This is especially important in medical diagnostics where confidence in prediction can influence clinical decision-making.

This graph in Fig. 17 represents a calibration curve for the LightGBM (LGBM) model, used to assess the reliability of predicted probabilities. The x-axis displays the mean predicted value, while the y-axis shows the fraction of positive outcomes. The dotted black line represents a perfectly calibrated model, where predicted probabilities accurately reflect real-world likelihoods. The blue line with square markers illustrates the calibration of the LGBM model, which deviates significantly from the ideal calibration. For instance, at a mean predicted probability of approximately 0.2, the fraction of positives is 1.0, which is excessively high, suggesting that the model overestimates probabilities in this range. Conversely, at a mean predicted probability of about 0.4, the fraction of positives drops to 0.0, indicating underestimation in this region. The inconsistencies in the curve highlight that the model’s probability outputs are unreliable and require recalibration to align closer to actual outcomes.

Figure 18 is a calibration curve for a stacked model, illustrating how well the model’s predicted probabilities align with actual outcomes. A dotted black line signifies perfect calibration, where predicted probabilities match real-world occurrences precisely. The solid blue line, representing the stacked model’s calibration, closely follows the ideal calibration line, indicating that the model produces reliable probability estimates. The accuracy of this calibration curve suggests that the stacked model effectively predicts probabilities without significant over- or underestimation.

A **Precision-Recall (PR) Curve** is a graphical tool used to evaluate the performance of a classification model, particularly in scenarios where the dataset is **imbalanced**—meaning the number of positive cases is much smaller than the number of negative cases (common in medical diagnosis tasks).

**Precision (Positive Predictive Value)**

Precision measures how many of the predicted positive cases are actually true positives.

10 $$Precision = \frac{{True\;Positives\left( {TP} \right)}}{{True\;Positives\left( {TP} \right) + False\;Positives\left( {FP} \right)}}$$

A high precision means the model makes few false positive errors.

**Recall (Sensitivity / True Positive Rate)**

Recall measures how many of the actual positive cases were correctly identified.

11 $$Recall = \frac{{True Positives \left( {TP} \right)}}{{True Positives \left( {TP} \right) + False Negatives \left( {FN} \right)}}$$

The **PR curve** plots **Precision** on the Y-axis and **Recall** on the X-axis, showing how the precision-recall tradeoff changes at different classification thresholds. A **high area under the PR curve (AUC-PR)** indicates both high precision and recall across thresholds. Unlike the ROC curve, which can present an overly optimistic view on imbalanced datasets, the PR curve focuses solely on the performance for the **positive class** and is more informative in such cases.

**Fig. 19:**
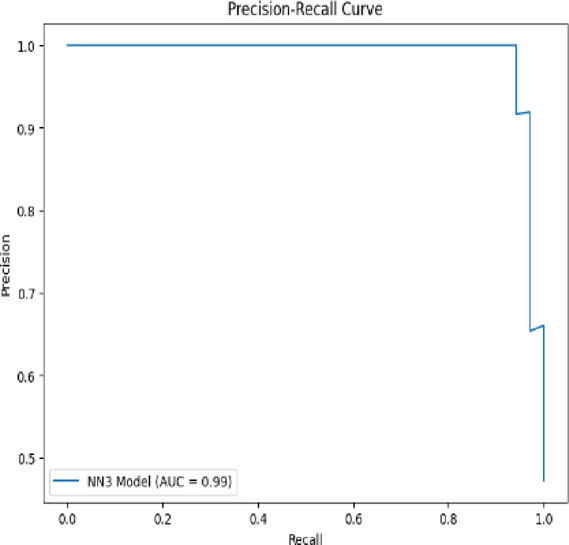
PR curve for TwinNet.

Figure 19 displays the **Precision-Recall (PR) curve in** TwinNet, a crucial evaluation for imbalanced classification problems, such as disease detection, where false negatives are costlier than false positives. This PR curve shows a near-perfect performance, with a steep drop only at the far end of recall, suggesting that for most thresholds, the model maintains very high precision along with high recall. The reported AUC of 0.99 further confirms this strong performance. It implies that the model has an exceptional ability to identify true positives without raising false alarms—a highly desirable trait in medical diagnosis models.

**Fig. 20:**
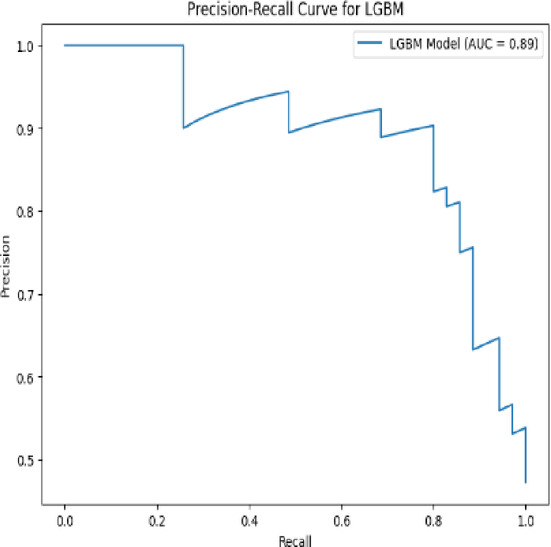
PR curve for LGBM model.

Figure 20 represents a Precision-Recall curve for a LightGBM (LGBM) model, providing a detailed analysis of the model’s classification performance. The y-axis denotes Precision, which measures the proportion of correctly identified positive instances among all predicted positive instances, while the x-axis represents Recall, which quantifies the proportion of correctly identified positive instances among all actual positive cases. Initially, at a recall of 0.0, the precision is at its peak (1.0), signifying perfect accuracy when no positive predictions are made. As recall increases, precision gradually declines due to the trade-off between capturing more positive instances and maintaining accuracy in predictions. The curve exhibits multiple steps, reflecting precision fluctuations at various recall levels. The Area Under the Curve (AUC) of this Precision-Recall plot is 0.89, indicating a strong model performance, as higher AUC values suggest better precision-recall balance. This graph is particularly useful for evaluating the LGBM model’s efficiency in handling imbalanced datasets, making it an essential tool for applications such as fraud detection, medical diagnosis, and recommendation systems where accurate classification is critical.

**Fig. 21:**
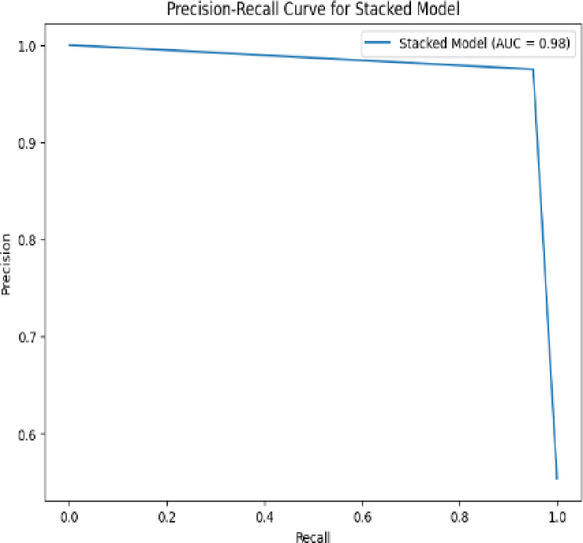
PR curve for Stacked model.

Figure 21 represents a precision-recall curve for a stacked model, with an impressive area under the curve (AUC) of 0.98. The precision-recall curve is crucial for evaluating the performance of classification models, particularly when dealing with imbalanced datasets. The curve begins at a precision of 1.0 and a recall of 0.0, meaning perfect precision is achieved when no positive instances are predicted. As recall increases, precision remains high before experiencing a sharp decline around a recall of 0.9. The high AUC value of 0.98 suggests that the stacked model is highly effective at distinguishing between positive and negative classes, maintaining strong precision and recall throughout most of the curve. This type of graph is particularly relevant for assessing how well the model handles imbalanced data while making accurate predictions, which is essential in real-world applications such as medical diagnostics, fraud detection, and natural language processing.

The **Accuracy Curve** plots the overall classification accuracy across different thresholds. Accuracy quantifies the ratio of correct predictions to total predictions and is given by:

12 $$Accuracy = \frac{{TP + True Negatives \left( {TN} \right)}}{{TP + TN + False Positives \left( {FP} \right) + FN}}$$

This curve is useful for selecting a threshold that optimizes overall correctness.

The **ROC Curve** (Receiver Operating Characteristic Curve) illustrates the trade-off between the **True Positive Rate (TPR)** and **False Positive Rate (FPR)** at different threshold settings. The formulas are:

13 $$TPR \left( {Recall} \right) = \frac{TP}{{TP + FN}}$$

14 $$FPR = \frac{FP}{{FP + TN}}$$

A perfect model has a curve that rises sharply towards the top-left corner. The area under the curve (AUC-ROC) is often used to summarize performance.

The **Loss Curve** tracks the model’s learning performance over training epochs. It typically plots both training and validation loss to monitor underfitting or overfitting. For binary classification, **Binary Cross-Entropy Loss** is commonly used, computed as:

15 $$Loss = - \frac{1}{N}\mathop \sum \limits_{i = 1}^{N} \left[ {y_{i} \log \left( {p_{i} } \right) + \left( {1 - y_{i} } \right)\log \left( {1 - p_{i} } \right)} \right]$$

where $y_{i}$ is the true label and $p_{i}$ is the predicted probability. A well-trained model will show decreasing training and validation losses that converge.

**Fig. 22:**
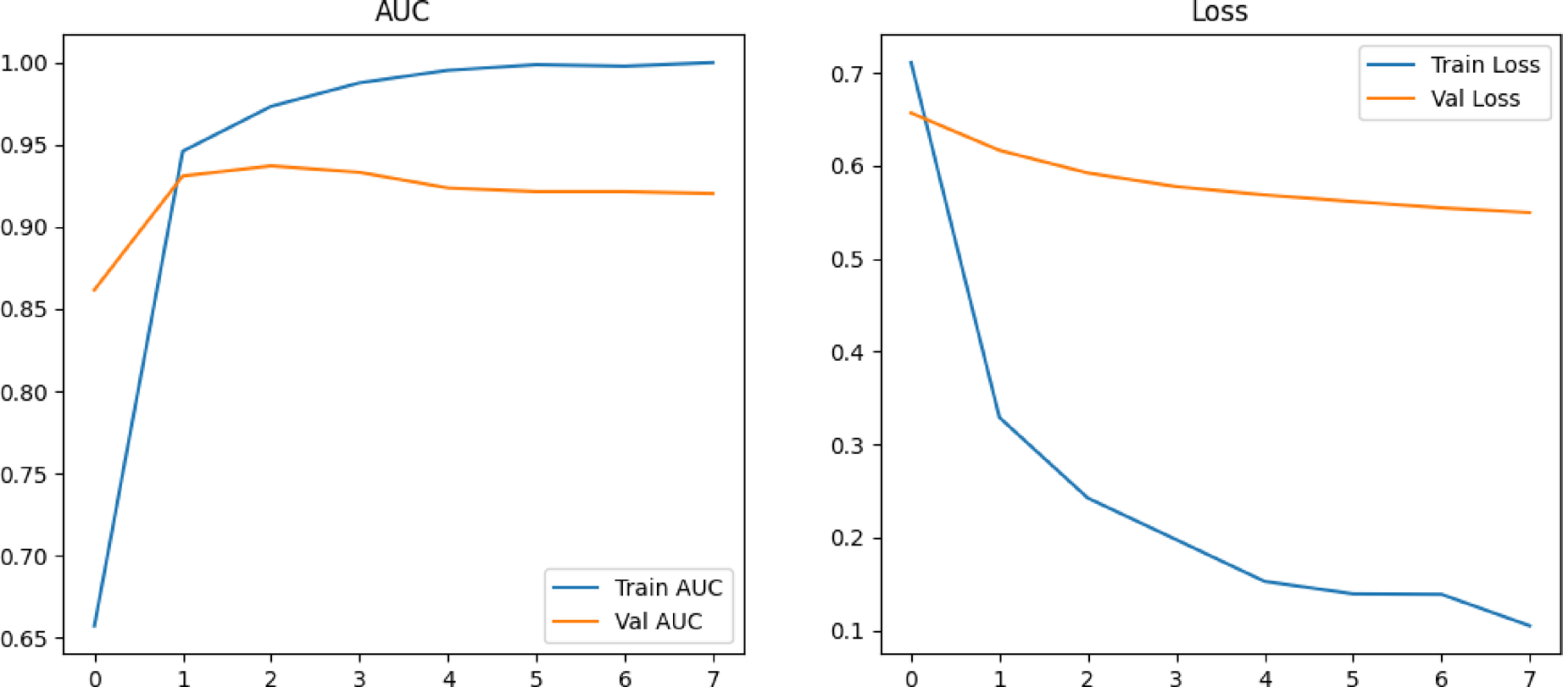
ROC_AUC curve and Loss curve for TwinNet.

The ROC_AUC curve and Loss curve for TwinNet is shown in Fig. 22 is shown below;

Figure 22 showcases two critical learning curves: the AUC curve (left) and the loss curve (right). The AUC (Area Under the Curve) graph plots both the training and validation AUC scores across epochs. Initially, the model’s training AUC increases sharply, indicating that the model rapidly learns meaningful patterns from the training data. This is followed by a more gradual improvement and stabilization at around 0.99, which signifies excellent discriminative power on the training set. In parallel, the validation AUC also improves and remains relatively stable around 0.92–0.93. This minor gap between training and validation AUC suggests the model generalizes well and is not severely overfitting. On the right, the loss curve presents a typical trend where training loss decreases steadily and significantly across epochs, while validation loss decreases more slowly and plateaus. The widening gap between training and validation loss may hint at slight overfitting, but not enough to compromise model validity.

**Sensitivity** measures the proportion of actual positive cases (i.e., patients who truly have the condition) that are correctly identified by the model or diagnostic tool. It reflects the test’s ability to **detect disease when it is present**, minimizing false negatives.

16 $$Sensitivity = \frac{{True\;Positives \left( {TP} \right)}}{{True\;Positives\left( {TP} \right) + False\;Negatives\left( {FN} \right)}}$$

A high sensitivity indicates that the model is effective in identifying most patients who have the condition. This is particularly important in screening tests where, missing a true case can have severe consequences (e.g., failing to detect a heart arrhythmia).

**Specificity**, on the other hand, measures the proportion of actual negative cases (i.e., patients who do **not** have the condition) that are correctly identified as negative. It assesses the test’s ability to **exclude disease when it is absent**, minimizing false positives.

17 $$Specificity = \frac{{True\;Negatives\left( {TN} \right)}}{{True\;Negatives\left( {TN} \right) + False\;Positives\left( {FP} \right)}}$$

A high specificity means that the model is accurate in confirming healthy individuals as disease-free. This is crucial when false positives may lead to unnecessary anxiety, additional testing, or treatment. Both metrics are essential in evaluating the clinical reliability of diagnostic tools. A **sensitive** test is good for **ruling out** disease, while a **specific** test is good for **ruling in** disease. In practical applications, a balance between sensitivity and specificity is often sought, depending on the clinical context and consequences of errors.

### Comparative analysis

Table 4 compares the result of well-known machine learning algorithm, existing models by authors^63–65^ and the proposed TwinNet model, stacked model. The results from the table below clearly shows that the proposed model shows better accuracy, specificity and sensitivity result. Thus, making TwinNet model, a better classification model.

**Table 4:** Comparative analysis between existing machine learning models and proposed classification model for CVD classification.

Models	Accuracy (%)	Specificity (%)	Sensitivity (%)
K-nearest neighbour^61^	81.48	77.37	85
Support vector machine^61^	83.84	78.83	81
Artificial neural network^61^	85.86	84	87.5
Naïve Bayes^61^	84.51	81	87.5
Voting classifier^62^	67	NA	NA
Gradient boosting^62^	70	NA	NA
Three phase ANN diagnostic system^63^	88.89	NA	NA
ANN_fuzzy_AHP diagnosis system^64^	91.1	NA	NA
FCMIM-SVM^65^	92.37	NA	NA
Light gradient boosting method	88	80	85
TwinNet (Proposed)	93.2	94	91.5
Stacked LGBM + TwinNet	91	95	85.3

### Complexity analysis

From Tables 5 and 6, it can be inferred that LightGBM relies on decision trees, which use conditional splits. Its computational complexity is based on the number of trees, depth, and leaves—this results in fewer operations and no need to store floating-point weights like NNs. TwinNet is a deep neural network with nearly 49,000 trainable weights, significantly more resource-intensive than LightGBM. Stacked model combines both LightGBM and TwinNet, making it the most complex, with the sum of both models’ costs and a small meta-learner.

**Table 5:** Complexity analysis of the designed models.

Model	Core idea	Train/infer cost	Capacity indicator	Inference
LightGBM	200 gradient-boosted trees (depth 10, 20 leaves each)	0.06 s to fit, ≈ O(1.48 × 105) ops per full prediction pass	≈ 40 k split nodes (implicit parameters)	Very light-weight; tree ensembles excel on tabular data and capture non-linear feature interactions quickly. With depths capped at 10 the risk of over-fitting is limited, yet the model still memorises fine-grained thresholds
TwinNet (custom NN)	4 dense blocks + BN (256–128-64–32) feeding a sigmoid head	minutes to fit (GPU) but only a few 100 µs to infer; 48 769 trainable weights	Parameter count directly equals complexity	The network learns distributed, continuous feature representations that trees cannot. Batch-norm layers stabilise training; the depth allows hierarchical abstraction. However, every extra neuron increases memory and FLOPs, so deployment is little costly
Stacked ensemble	OOF meta-features from LightGBM + TwinNet → logistic-regression blender	train cost ≈ cost(LGBM) + cost(NN) + tiny blender; inference cost is cumulative; complexity ≈ 1.97 × 105 ops	Effective capacity ≈ parameters(NN) + split-nodes(LGBM) + 3 blender weights	By letting the meta-learner weigh complementary error patterns, the stack almost doubles raw model complexity relative to the NN alone, yet remains tractable. It usually pushes a few points of ROC-AUC/accuracy further, at the expense of twice the RAM and an inference path that now calls two base learners, latency, is the bottleneck

**Table 6:** Complexity metric

Model	Complexity metric	Value
LightGBM	Approximate time complexity	O(148,000)
TwinNet NN	Number of trainable parameters	48,769
Stacked	Combined (LGBM + NN + meta-learner)	O(196,771)

## Challenges in proposed methodology

Developing a patient digital twin poses several interrelated challenges. First, data quality and accuracy are critical, as the twin gathers data from various sources such as EHRs and wearable sensors, all of which must be precise and harmonized into a consistent format—something that is difficult in practice due to missing, noisy, or inconsistent data. Secondly, the process is both complex and costly, requiring advanced software development, continuous monitoring, and considerable investment in computational infrastructure and specialized expertise in healthcare modelling and data science^66^. Another major hurdle lies in integrating the digital twin with existing systems like EHRs and HIEs (Health Information Exchange), which demands interoperability through standardized data formats, communication protocols, and workflows.

Furthermore, accurately modelling physiological systems such as the cardiovascular network is inherently difficult because human systems are dynamic, vary significantly between individuals, and exhibit complex interdependencies across bodily systems. Each person has unique genetic makeup, medical history, and lifestyle factors (e.g., diet, exercise, stress levels) that influence how their physiological systems function. This variability makes it difficult to create a one-size-fits-all model that can accurately represent the behaviour of every individual’s body. Another important thing to note is that physiological systems in the body do not function in isolation^67^.

The cardiovascular system, respiratory system, nervous system, and others are deeply interconnected. A change in one system can affect multiple other systems, and accurately simulating these interactions requires highly sophisticated models that can account for these cross-system dependencies. Finally, real-time data integration adds another layer of complexity, as continuous streams from diverse sources must be harmonized and processed efficiently to maintain simulation accuracy posing significant demands on both data quality management and computational resources. Moreover, gaps in data (e.g., missing vital signs or incomplete patient histories) can hinder the accuracy of the physiological model^68^. Collecting data from diverse sources (e.g., IoT devices, lab results, medical records) and framing it into a single, coherent dataset that can be fed into a model is a significant hurdle in accurate patient twin modelling.

## Conclusion

The TwinCardio is a smart cardiovascular health monitoring digital twin model that intends to provide an individualized monitoring tool and diagnostic tool and generate a personalized treatment plan for the patient by understanding the function of the patient’s heart using an echocardiogram chest sensor. The on-body sensor implanted on patient’s chest is an echo sensor used to generate the echo images of the heart and send it in the form of a video file which will be further used to generate a 3D visualization model of the heart, giving a clear image of the status of the heart. The video, along with the 3D model and the pulse rate captured by the sensor, will serve as the data collected from the patient.

The reference framework proposed for the cardiovascular digital twin model and its various layers uses the patient-specific data gathered by the sensor, the digital twin will serve as an advanced tool that will help the physician to visualize, diagnose, and customize treatment plans for the patient. As it is not possible to get real-time sensor data from a patient, the paper has taken the echo dataset from the online available Stanford University “Echo-Net Dynamic” dataset as the video data set and used the same to gnerate a 3D visualization image. Since digital twin technology is still in its infancy in the healthcare sector, it is quite challenging to produce real-time simulation data. This work serves as a reference framework for the digital twin model in the field of cardiovascular disease diagnosis. Further work is being carried out to generate some viable results to support this framework.

Second half of the paper discusses different classification models like Light Gradient Boosting Method (LGBM), custom designed neural network—TwinNet and a stacked ensemble model, that combines TwinNet and LGBM model. Out of all the three, TwinNet gives better result and therefore, is proposed to be used for CVD classification and prediction in designing digital twin model in our future work. This classification and prediction method will act as a first layer to understand the patient details, depending on which; simulation model can be modified. Also, a comparison between existing classification model and proposed TwinNet classification model is also shown.

The integration of digital twin technology with healthcare will prove to be a boon for this sector, as digital twin not only provides real-time analysis and personalized treatments but also aids in predictive analysis. With the help of digital twin technology, current data acquired from the echo sensor, as well as the past record, a prediction model is developed that will help to forecast any possible heart ailments. Anomaly detection is another essential component of the digital twin approach. It triggers an alarm in real-time if there are any significant deviations from the normal heart activity. Digital twin in healthcare is still an up-and-coming technology and still necessary research is being carried out to identify possible applications of the same. Overall, the proposed TwinCardio and TwinNet model offers a strong, comprehensive, and customized method of cardiovascular health monitoring.

## Data Availability

The authors are willing to share the experiment data to the interested researchers on request to the corresponding author.
